# The 4-alkyl chain length of 2,5-dimethoxyamphetamines differentially affects in vitro serotonin receptor actions versus in vivo psychedelic-like effects

**DOI:** 10.1038/s41380-025-03325-1

**Published:** 2025-11-05

**Authors:** Dino Luethi, Grant C. Glatfelter, Eline Pottie, Francesca Sellitti, Alexander D. Maitland, Nicholas R. Gonzalez, Lindsay A. Kryszak, Shelley N. Jackson, Marius C. Hoener, Christophe P. Stove, Matthias E. Liechti, Martin Smieško, Michael H. Baumann, Linda D. Simmler, Deborah Rudin

**Affiliations:** 1 Psychopharmacology Research, Department of Biomedicine, University Hospital Basel and University of Basel, Basel, Switzerland; 2 Designer Drug Research Unit, National Institute on Drug Abuse Intramural Research Program, Baltimore, MD US; 3 Laboratory of Toxicology, Department of Bioanalysis, Faculty of Pharmaceutical Sciences, Ghent University, Ghent, Belgium; 4 Neuropharmacology, Department of Pharmaceutical Sciences, University of Basel, Basel, Switzerland; 5 Translational Analytical Core, National Institute on Drug Abuse Intramural Research Program, Baltimore, MD US; 6 Neuroscience Research, pRED, Roche Innovation Center Basel, F. Hoffmann-La Roche Ltd, Basel, Switzerland; 7 Computational Pharmacy, Department of Pharmaceutical Sciences, University of Basel, Basel, Switzerland; 8 Present Address: Société des Produits Nestlé, Nestlé Research, Lausanne, Switzerland

**Keywords:** Neuroscience, Molecular biology

## Abstract

Various ring-substituted α-methylphenethylamines (i.e., amphetamines) produce psychedelic-like effects that are primarily mediated by activity at 5-hydroxytryptamine 2A (5-HT_2A_) receptors. Small lipophilic substituents at the 4-position of the 2,5-dimethoxyamphetamine core structure can greatly enhance the clinical potency of such derivatives. Here, we studied the effects of various 4-alkylated 2,5-dimethoxyamphetamines (4-methyl, 4-ethyl, 4-propyl, 4-butyl, 4-amyl) on in vitro receptor activities and in vivo psychedelic-like effects in mice. The acute effects of the compounds were examined using the mouse head-twitch response (HTR) assay, a proxy for psychedelic-like drug actions. Overall, the series primarily interacted with 5-HT_2_ receptor subtypes, with increasing 4-alkyl chain length associated with increased affinity at 5-HT_2A_ receptors. For all three in vitro functional readouts assessed, the 4-propyl analog produced the highest potencies for 5-HT_2A_ receptor activation (1–9 nM), but smaller and longer chain lengths displayed comparable activities (2–56 nM). In mice, the compounds displayed variable maximal HTR counts (23–119) and potencies (0.42–2.76 mg/kg), with the 4-propyl and 4-methyl compounds being the most potent and efficacious, respectively. Analysis of drug concentrations in mouse plasma, brain tissue, and brain dialysate samples revealed that derivatives with longer alkyl chains (i.e., butyl, amyl) require higher systemic doses to achieve concentrations comparable to those of short-chain analogs. These findings demonstrate that extending the 4-position alkyl chain beyond a propyl group reduces in vivo potency and efficacy, in part due to pharmacokinetic parameters.

## Introduction

Many derivatives of phenethylamine are potent 5-hydroxytryptamine 2 (5-HT_2_) receptor ligands with therapeutic potential and usefulness in serotonergic systems research [1–3]. In addition, a variety of substituted phenethylamines have appeared on the recreational drug market due to their reported psychedelic and euphoric properties [4, 5]. Even though the risk of physical harm from use of phenethylamine psychedelics is low [4–7], overdose can result in a variety of adverse effects [4, 5, 8].

Psychedelic phenethylamines, and other classes of psychedelics such as lysergamides and tryptamines, act as partial to full agonists at the 5-HT_2A_ receptor subtype [9–16] and mediate 5-HT_2A_-dependent behaviors in vivo, such as the head-twitch response (HTR) in mice [17–25]. A recent study suggests that the HTR in mice is related to 5-HT_2A_-G_q_ efficacy of a given drug, whereas β-arrestin 2-biased agonists block psychedelic-like effects and can induce receptor downregulation [26]. Based on the aforementioned study, it has been postulated that a certain threshold level of 5-HT_2A_-G_q_ activity is required to achieve psychedelic-like effects, and low efficacy agonists do not elicit HTRs [26]. In addition to 5-HT_2A_ agonism, most phenethylamine psychedelics are agonists at 5-HT_2B_ and 5-HT_2C_ receptors [2]. Agonist actions at 5-HT_2B_ receptors may pose risk for cardiac mitogenesis and valvulopathy when psychedelics are taken chronically, such as repeated microdosing [27], but more clinical studies are needed to address this issue. Agonist actions at 5-HT_2C_ receptors can functionally oppose expression of 5-HT_2A_-mediated behavior after high drug doses, which contributes to the biphasic dose–response curves exhibited for HTR and locomotion in mice [2, 28–30]. On the other hand, some evidence indicates that 5-HT_2C_ activity is required for psychedelic-like effects, including HTR [30, 31].

Many 5-HT_2A_ agonists also interact with 5-HT_1A_ receptors, and this activity modulates pharmacological effects of the drugs. For example, 5-HT_1A_ receptors are colocalized with 5-HT_2A_ receptors on cortical pyramidal neurons, where the receptor types exert opposing effects [2, 32, 33]. Various tryptamine psychedelics have agonist activity at 5-HT_1A_ receptors that can dampen their psychedelic-like effects in rodents and humans [24, 34–41]. In general, phenethylamine psychedelics are more selective for 5-HT_2A_ vs. 5-HT_1A_ receptors [4, 36, 42, 43], though their psychedelic-like effects can still be modulated by 5-HT_1A_ agonism in some cases [43, 44].

**Fig. 1:**
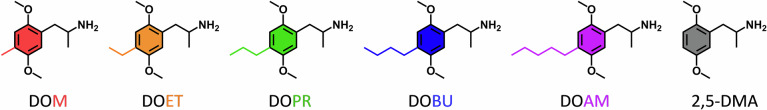
Structures of 4-alkylated 2,5-dimethoxyamphetamine (2,5-DMA) derivatives. “DO” stands for desoxy, and last letters indicate the alkyl chain: methyl, ethyl, propyl, butyl, or amyl.

Other potentially relevant targets of psychedelics include adrenergic, dopaminergic, histaminergic, or opioid receptors, as well as monoamine transporters and monoamine oxidases [4, 5, 9, 11, 14, 15, 34, 45–47]. Basic questions remain regarding how different structural features of psychedelic phenethylamines influence their comparative receptor target profiles, psychedelic subjective effects, and potential therapeutic utility [4, 5, 48, 49]. Here, we studied a series of 4-alkylated derivatives of 2,5-dimethoxyamphetamine (2,5-DMA; Fig. 1) using in vitro and in vivo pharmacological methods. These derivatives were originally synthesized by the medicinal chemist Alexander Shulgin, through systematic structural modifications of mescaline (3,4,5-trimethoxyphenethylamine) [48]. Alpha-methylation of mescaline yielded 3,4,5-trimethoxyamphetamine (TMA), and subsequent rearrangement from a 3,4,5 to a 2,4,5-trimethoxy group (2,4,5-trimethoxyamphetamine; TMA-2) increased the potency around one order of magnitude [48, 50]. Replacement of the 4-methoxy group with a methyl group further increased the potency of the respective derivative “desoxy-TMA-2” (i.e., DOM) an additional 5–10-fold, with efficacious human doses being in the range of 3–10 mg [48].

Interestingly, 2,5-DMA is devoid of robust psychoactive effects [48], while its more potent 4-methyl derivative DOM was a popular recreational drug in the late 1960s under the street name “STP” [48, 51]. Among the 4-alkylated derivatives of 2,5-DMA, the reported psychoactive effects are optimal for two- and three-carbon chain lengths (DOET and DOPR, respectively), with decreased effects for four-carbon and five-carbon chain lengths (DOBU and DOAM, respectively) [52]. Enantioselective activity has been reported for 2,5-DMA derivatives, with the R isomers exhibiting higher activity in animals and humans [43, 48]. Here, we aimed to further study the structure–activity relationships of 4-alkylated 2,5-DMA derivatives to better understand the relationship between receptor interactions in vitro and psychedelic-like behavioral effects in vivo. Specifically, we studied three signaling cascades linked to 5-HT_2A_ receptor activation as well as interactions with other serotonergic and non-serotonergic targets. Furthermore, we assessed the HTR in mice as a behavioral proxy for psychedelic-like effects and quantified drug concentrations in post-mortem plasma and brain tissue. To assess extracellular drug concentrations in the brain, we conducted in vivo microdialysis experiments.

## Materials and methods

### Drugs

2,5-Dimethoxyamphetamine hydrochloride (2,5-DMA HCl), 4-methyl-2,5-dimethoxyamphetamine (DOM), 4-ethyl-2,5-dimethoxyamphetamine (DOET), 4-propyl-2,5-dimethoxyamphetamine hydrochloride (DOPR HCl), 4-butyl-2,5-dimethoxyamphetamine (DOBU), and 4-amyl-2,5-dimethoxyamphetamine (DOAM) were obtained from Cayman Chemical (Ann Arbor, MI, USA). All substances were obtained as racemic mixtures with > 98% purity. 5-HT was purchased from Merck (Buchs, Switzerland).

### Receptor and transporter affinity

Binding affinities at human monoamine receptors and transporters were assessed as previously reported [15]. A detailed description of the methodology is provided in the Supplementary information.

### Functional activity at human 5-HT receptors

Activation of h5-HT_2A_, h5-HT_2B_, and h5-HT_2C_ receptors was examined by measuring the accumulation of inositol monophosphate (IP_1_) as a marker for Gα_q_-mediated signaling. For h5-HT_2A_, additional assays measured β-arrestin 2 recruitment and arachidonic acid release, the latter serving as a marker of Gα_i/o,12/13_ activation. The methodology is described in detail in the Supplementary information.

### Functional activity at the human TAAR1

Activity at the human trace amine-associated receptor 1 (hTAAR1) was determined as previously described [53]. More information can be found in the Supplementary information.

### In silico studies

3D docking was performed using well-resolved structures of the 5-HT_2A_ and 5-HT_2C_ receptor. To account for receptor conformational variability and enhance the robustness of the docking results, three distinct crystal structures of the 5-HT_2A_ receptor were used, each representing a different conformational state observed under varying crystallographic conditions (PDB ID: 7WC4 [54], 7WC8 [54], 9AS8 [55]); only one structure was used for the 5-HT_2C_ receptor (PDB ID: 8DPF [55]). In addition, pharmacokinetic and physicochemical properties were predicted with the QikProp tool (Schrödinger, New York, NY, USA). More details about molecular modeling can be found in the Supplementary information.

### Behavioral studies

Behavioral studies were conducted as previously described with minor modifications [23, 24, 36]. Male C57BL/6 J mice (The Jackson Laboratory #000664, 24 mice/drug) were purchased at 8 weeks old, group housed for acclimation (3–5 per cage for 1–2 weeks) under a 12:12 light:dark cycle (07:00 local time = lights on), with *ad libitum* access to food and water at the NIDA IRP facilities in Baltimore, MD, USA. All experiments were approved by the NIDA IRP Animal Care and Use Committee. After acclimation to the vivarium facilities, each mouse was briefly anesthetized with isoflurane and a temperature transponder (14 × 2 mm, model IPTT-300, Bio Medic Data Systems, Inc., Seaford, DE, USA) was implanted subcutaneously (s.c.) on the back, posterior to the shoulder blades. After the implant, mice were single housed and allowed one week recovery prior to the start of experiments. DOM, DOBU, and DOAM were dissolved in DMSO/Tween80/saline vehicle (1:1:18), while the hydrochloride salts of DOPR and 2,5-DMA were dissolved in saline vehicle. Drugs were randomly assigned and administered s.c. as the weight of the form used at an injection volume of 0.01 mL/g body weight.

During experimental testing, mice were tested once every 1–2 weeks to avoid potential tolerance to behavioral effects of the test drugs [43, 56] and were tested 2–3 times in total per drug. Each test session began with a brief 5 min acclimation period to the testing arenas, followed by recording baseline body temperature using a handheld wand that was sensitive to the signals emitted from the s.c. transponder. After this, mice were injected s.c. with appropriate doses of test drug and returned to the chambers for a 30-min test session. During the dose-response sessions, locomotor activity was continuously recorded using open field chambers with photobeam arrays (Coulbourn Instruments, Holliston, MA, USA) that were modified with cylindrical inserts and custom floor panels that also allowed video recordings of each mouse to later quantify HTR activity (GoPro Hero 7 camera; 960p resolution at 120 frames per second). Videos for each mouse were analyzed post session using commercially available software used to quantify HTR as previously described [23].

### Analysis of brain and plasma drug concentrations

#### Animals and behavior

C57BL/6 J mice (60 total, 10/group, 5 male and 5 female/group, Jackson Labs) were used to conduct behavioral experiments after administration of single s.c. doses of DOM (3 mg/kg), DOPR (1 mg/kg), DOBU (3 mg/kg), DOAM (10 mg/kg), 2,5-DMA (10 mg/kg), or DMSO/Tween80/saline vehicle (1:1:18) as described in Section 2.6. In these experiments, HTR activity was monitored for 20 min, followed by cardiac puncture and decapitation. Whole blood (~ 300–500 µL per sample) was collected via cardiac puncture, placed in tubes containing 10 µL of heparin (1000 IU/mL), and immediately stored on ice until centrifugation. Blood samples were spun at 3300 rpm at 4 °C and plasma was decanted into vials on dry ice until storage at -80 °C. Whole brains were collected immediately after cardiac puncture. Brains were frozen on dry ice, weighed, then placed into 7-mL homogenizing tubes with ceramic beads, and stored at -80 °C.

#### Bioanalysis

A detailed description of the sample preparation and bioanalysis is provided in the Supplementary information.

### In vivo microdialysis

Microdialysis experiments were conducted as previously reported [57] with minor modifications; involved procedures were approved by the Cantonal Veterinary Office Basel-Stadt, in accordance with Swiss law. Three male and two female C57BL/6JRj mice (Janvier Labs, France) aged 11–14 weeks were used. During stereotactic surgeries, a guide cannula (CMA7; CMA Microdialysis AB, Sweden) directed to the striatum was implanted at 0.6 AP, 1.8 ML and -1.5 DV from bregma and fixed with dental cement. On the day of microdialysis, a microdialysis probe (CMA7, 3-mm probe length) was inserted into the guide cannula and perfused with artificial cerebrospinal fluid (119 mM NaCl, 11 mM D-glucose, 26.2 mM NaHCO_3_, 2.5 mM KCl, 1.3 mM MgCl_2_, 1 mM NaH_2_PO_4_, and 2.5 mM CaCl_2_) at 1 µL/min. The dialysate was collected for 30 min before s.c. injection of a mixture of DOM and DOAM (dissolved in DMSO/Tween80/saline; 1:1:18) yielding a dose of 0.5 mg/kg body weight for each substance. The dialysate post injection was collected for 30 min followed by decapitation under isoflurane anesthesia. Plasma obtained from trunk blood and homogenates of the forebrains (0.5 g brain tissue per 1 mL ice-cold water) were prepared for LC–MS/MS analysis. One hemisphere of the brain from each mouse was fixed in 4% paraformaldehyde, sectioned, and stained with Hoechst dye (2 µg/mL for 30 min) to confirm the placement of the microdialysis probe in the striatum. After running microdialysis in one or two mice, the recoveries of the microdialysis probes were assessed by collecting dialysate for 15 min at 1 µL/min with the probe immersed in a mixture of DOM and DOAM (1 µg/mL each; in artificial cerebrospinal fluid).

### Data analysis

Data analysis was performed with Prism software (version 10.1.2, GraphPad Software, Boston, MA, USA). The IC_50_ values from the radioligand binding assays were determined by nonlinear regression, using three independent concentration-response curves fitted to a one-site model. The Cheng-Prusoff equation was used to calculate *K*ᵢ values for the competing ligands. EC_50_ values were determined by applying nonlinear regression to concentration-response curves. Bias factors (β) were calculated based on the relative intrinsic activity approach [58, 59]. More detailed information can be found in the Supplementary information. Mean values for total number of HTR, change in body temperature from pre to post session (Δ°C), and total distance traveled (cm) for each drug dose were compared (*p* < 0.05*)* via Welch’s ANOVA with Dunnett’s T3 post test, comparing all doses to respective vehicle controls (0 mg/kg). Welch’s ANOVA with Dunnett’s T3 post test was also used to compare drug concentrations, but the data were compared to DOM in this case. Dose–response relationships for HTR and locomotor activities were plotted using bell-shaped curve fits, while temperature change data were plotted using four-parameter non-linear regression curve fits. The ascending phase of the HTR curve for each drug was used to determine ED_50_ potency values from four parameter non-linear regression fits. For combined DOM and DOAM administration, the plasma, brain tissue, and brain dialysate drug concentrations were compared using paired t-tests (*p* < 0.05). Ratios were compared using Holm-Sidak’s post hoc test following a repeated-measures one-way ANOVA (F (1.709, 6.835) = 70.80; *p* < 0.0001).

## Results

### Interactions with serotonin 5-HT receptors

#### Binding affinities

Binding affinities of the DOM analogs at 5-HT_2_ and 5-HT_1A_ receptors are shown in Table 1. 2,5-DMA did not display relevant (i.e., submicromolar) affinity at any of the tested 5-HT receptors. However, addition of a small lipophilic methyl substituent at the 4-position markedly increased the affinity of the compound at 5-HT_2_ receptors (*K*_i_ = 66–404 nM) without a distinct effect on 5-HT_1A_ receptor affinity (*K*_i_ = 7.2 µM). The 5-HT_2A_ affinity was enhanced with increasing length of the 4-alkyl chain, whereas no clear trend was observed for the other 5-HT_2_ receptors for this series.

**Table 1:** Interactions of 4-alkylated 2,5-dimethoxyamphetamines with serotonergic receptors.

	DOM	DOET	DOPR	DOBU	DOAM	2,5-DMA
*Receptor binding*						
*K*_i_ h5-HT_1A_ [nM] (95% CI)	7238 (5082–10,292)	5761 (3911–8447)	3951 (2627–5919)	13,530 (8691–21,364)	8424 (5393–13,021)	6017 (4387–8232)
*K*_i_ h5-HT_2A_ [nM] (95% CI)	66 (33–99)	12 (11–13)	11 (9–13)	5.4 (4.6–6.2)	3.5 (2.1–4.9)	2502 (2280–2720)
*K*_i_ h5-HT_2B_ [nM] (95% CI)	149 (92–245)	174 (98–323)	126 (75–217)	162 (95–279)	98 (45–211)	5184 (2407–9918)
*K*_i_ h5-HT_2C_ [nM] (95% CI)	404 (265–543)	108 (32–184)	47 (35–59)	60 (45–75)	75 (59–91)	> 5000
*Selectivity (binding ratios)*						
5-HT_2A_/5-HT_1A_	110	480	359	2506	2407	2
5-HT_2A_/5-HT_2B_	2	15	11	30	28	2
5-HT_2A_/5-HT_2C_	6	9	4	11	21	> 2
*IP*_*1*_ *formation*						
EC_50_ h5-HT_2A_ [nM] (95% CI)	21 (15–28)	12 (8–19)	1.9 (1.4–2.6)	2.8 (1.9–4.2)	8.1 (4.9–14.0)	1379 (1084–1779)
E_max_ h5-HT_2A_ [%] (95% CI)	92 (88–97)	93 (86–99)	96 (92–101)	90 (85–95)	83 (79–88)	99 (95–103)
EC_50_ h5-HT_2B_ [nM] (95% CI)	688 (348–1263)	236 (105–563)	228 (115–484)	570 (273–1052)	572 (257–1160)	93,320 (37,183–211,526)
E_max_ h5-HT_2B_ [%] (95% CI)	91 (79–105)	95 (82–110)	96 (86–107)	122 (105–140)	106 (90–124)	94 (76–114)
EC_50_ h5-HT_2C_ [nM] (95% CI)	3.2 (2.0–4.9)	0.57 (0.40–0.82)	0.93 (0.65–1.33)	0.13 (0.06–0.28)	0.90 (0.43–1.93)	124 (85–184)
E_max_ h5-HT_2C_ [%] (95% CI)	98 (91–105)	91 (85–96)	99 (93–105)	95 (88–103)	94 (83–105)	103 (98–109)
*β-arrestin 2 recruitment*						
EC_50_ h5-HT_2A_ [nM] (95% CI)	55 (42–71)	12 (9–15)	9.3 (6.8–12.6)	24 (15–38)	56 (34–89)	1658 (1215–2293)
E_max_ h5-HT_2A_ [%] (95% CI)	97 (93–100)	99 (96–103)	105 (101–110)	96 (88–104)	85 (77–93)	83 (79–88)
*Arachidonic acid release*						
EC_50_ h5-HT_2A_ [nM] (95% CI)	3.7 (2.8–5.0)	1.5 (1.0–2.0)	0.86 (0.55–1.35)	2.0 (1.7–3.4)	2.4 (1.8–3.2)	160 (95–265)
E_max_ h5-HT_2A_ [%] (95% CI)	75 (71–79)	88 (82–95)	117 (105–129)	74 (70–79)	91 (87–95)	59 (57–62)
*Bias factor β*^*1*^						
Gα_i/o,12/13_/Gα_q_	0.04	0.16	-0.53	-0.61	-0.30	0.37
β-arrestin 2/Gα_q_	-0.35	0.08	-0.64	-0.86	-0.73	0.09
Gα_i/o,12/13_/β-arrestin 2	0.39	0.07	0.11	0.25	0.43	0.29

^1^Positive bias factor values indicate a relative preference for pathway indicated in numerator over pathway indicated in denominator and vice versa.

#### Receptor activity

Activation potencies and efficacies of the tested DOM analogs at different 5-HT_2_ receptors are shown in Table 1. The potency to activate 5-HT_2A_ signaling as well as Gα_q_-mediated 5-HT_2B_ signaling was slightly enhanced by increasing 4-alkyl chain length up to three carbon atoms (i.e., DOET, DOPR) and then decreased or was equivalent to DOM (i.e., DOBU, DOAM). At the 5-HT_2C_ receptor, potencies for Gα_q_-mediated signaling were relatively similar (EC_50_ range = 0.13–3.2 nM) except for 2,5-DMA (EC_50_ = 124 nM), and alkyl chain length did not seem related to the observed functional activity. At the 5-HT_2A_ receptor, the majority of substances (DOM, DOET, DOPR, DOBU) were full agonists in the β-arrestin 2 recruitment assay, whereas most substances were high efficacy partial agonists for Gα_q_ (DOM, DOET, DOBU, DOAM) and Gα_i/o,12/13_-mediated signaling (DOM, DOET, DOBU, DOAM, 2,5-DMA).

#### 5-HT_2A_ receptor activation bias

The quantitative bias factors, calculated using 5-HT as the reference agonist, are presented in Table 1 and Fig. S1. Bias factors close to 0 imply no bias toward either tested signaling pathway. Positive values indicate a preference for pathway 1, and negative values indicate a preference for pathway 2, relative to the reference agonist 5-HT. Significance of the bias is indicated in Fig. S1. Overall, longer alkyl chains increased the bias toward Gα_q_-mediated signaling over β-arrestin 2 recruitment, whereas no other trend was observed (Fig. S1A–D).

### Interactions with other monoaminergic targets and TAAR1

Binding affinities of the DOM analogs at monoaminergic targets other than 5-HT receptors are shown in Table S1. 2,5-DMA and all 4-alkylated derivatives displayed weak affinities for binding at the α_1A_ receptor (*K*_i_ = 2.1–7.4 µM). Most of the compounds in the series exhibited low micromolar or no affinity at the α_2A_ receptor, except for DOPR (*K*_i_ = 513 nM). Further, DOAM displayed binding affinity at monoamine transporters (DAT and SERT, *K*_i_ = 3.6–4.6 µM), while no binding was observed for the other compounds at monoamine transporters in the investigated concentration range (*K*_i_ > 7 µM). Similarly, no binding at dopamine D_2_ receptors was measured for any of the compounds (*K*_i_ > 7.5 µM). DOPR, DOBU, and DOAM were the only ones to activate TAAR1 (EC_50_ of 10.1–21.8 µM) with 86–98% activation efficacy (Table S2).

### 3D docking experiments

Ligand docking studies at the 5-HT_2A_ and 5-HT_2C_ receptors suggest that the binding sites for all substances lie within the same region of the receptor, are composed of similar key amino acid residues, and feature similar multi-pocket shape and a rather large volume. They all contain an aspartate residue (Asp155) as the key “anchoring” residue capable of forming the salt bridge to the protonated amine group of the ligands, but the topology of the ligand-binding cavities does not further restrict the position of the studied small-molecule ligands (Fig. S2).

### In vivo psychedelic-like effects in mice

To examine the structure–activity relationship of 4-alkylated 2,5-DMA derivatives to produce psychedelic-like effects in vivo, the HTR was assessed in mice after acute drug administration. Locomotor activity and body temperature were also assessed to monitor other pharmacological effects of the drugs that may impact the HTR. Each compound was administered at doses in the range of 0.03–30 mg/kg over a 30 min testing session.

**Fig. 2:**
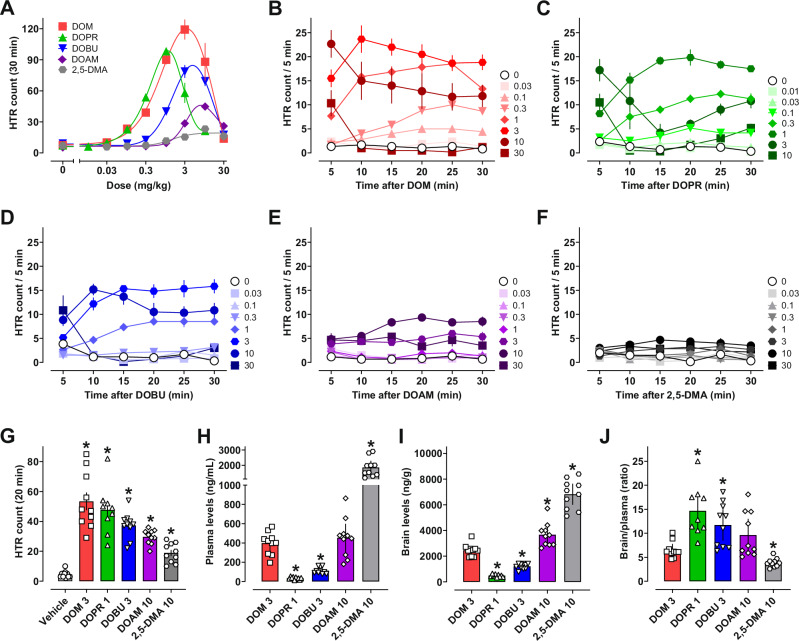
Acute psychedelic-like effects as well as brain and plasma concentrations of 2,5-DMA derivatives varying in 4-alkyl chain length. **A** Dose–response for total number of HTRs induced by each drug (*n* = 4–6 mice per dose). Time course of HTR events across the testing session for **B** DOM, **C** DOPR, **D** DOBU, **E** DOAM, and **F** 2,5-DMA. **G** HTR counts prior to brain and blood collection (*n* = 10 mice per drug), **H** plasma and **I** brain tissue levels, as well as **J** the drug level ratios. Values shown in panels **A**–**G** are means ± SEM, while panels **H–J** are means with 95% CI; further statistical information and exact sample sizes are provided in Table S3. Individual values for the dose–response curves in panel **A** are shown in Fig. S3.

The dose–response curves for mean total HTRs induced by 2,5-DMA, DOM, DOPR, DOBU, and DOAM over the testing session are shown in Fig. 2A and Fig. S3A–E, while the HTR time course plots are shown in Fig. 2B–F. All test drugs produced significantly higher HTR counts vs. vehicle controls at various doses noted in Table S3. DOM and DOPR induced the highest total number of twitches, which decreased with increasing length of the 4-alkyl chain, while 2,5-DMA induced the lowest total HTR count but was notably active relative to vehicle controls (Table 2). The rank order of potencies (ED_50_ = 0.42–2.76 mg/kg) to induce the HTR was DOPR ≥ DOM ≥ DOBU ≥ 2,5-DMA ≥ DOAM (Table 2). Some differences in the time course of HTR production were observed across the series. DOM reached peak HTR rate (24 events/5 min) between 5–10 min post drug administration, while all other compounds showed peak HTR rates (5–20 events/5 min) somewhat later, between 10–20 min post drug administration (Table 2, Fig. 2B–F). In summary, all compounds induced psychedelic-like effects, with short to intermediate 4-alkyl chain lengths (methyl, propyl) producing the most potent and efficacious compounds in this regard.

**Table 2:** Potencies of 2,5-DMA derivatives varying in 4-alkyl chain length for psychedelic-like effects in mice.

	HTR in mice			Human dose^a^
	ED_50_	E_max_	Maximum rate	
	[mg/kg] (95% CI)	[total HTR count]	[HTR count/5 min]	
DOM	0.66 (0.46–1.91)	119	24	3.0–10.0 mg
DOET^b^	0.20 (0.11–0.36)	122		2.0–6.0 mg
DOPR	0.42 (0.29–1.13)	98	20	2.5–5.0 mg
DOBU	1.29 (0.97–14.97)	79	16	Uncertain
DOAM	2.76 (2.21–4.19)	45	9	> 10 mg
2,5-DMA	1.39 (0.47–3.95)	23	5	80–160 mg

^a^Human doses are taken from *PiHKAL: A Chemical Love Story* [48].

^b^Data from Halberstadt et al. 2020 [20].

Body temperature change and locomotor activity were also monitored in conjunction with measuring HTR. Effects of 2,5-DMA and its 4-alkylated derivatives on mean body temperature change across the session are shown in Fig. S4A. All substances induced dose-dependent decreases in body temperature, with the highest potency displayed by DOPR. Dose-dependent effects of the test compounds on locomotor activity are shown in Fig. S4B, with time course data shown in Fig. S5A–E. Overall, small increases in locomotor activity were seen at lower doses (i.e., 0.3–10 mg/kg), while activity was generally decreased at higher doses (i.e., 10–30 mg/kg) compared to vehicle controls. Notably, the doses of the test compounds that reduced body temperature and locomotor activity were ~10–30-fold higher than HTR-active doses, showing that the compounds were much more potent for producing psychedelic-like effects vs. serotonin-syndrome-like effects.

### In silico prediction of blood–brain penetration

Blood–brain penetration parameters predicted with the QikProp tool are listed in Table S4. The predicted brain/blood partition coefficient (QPlogBB) indicates a gradually less favorable partitioning for the brain for derivatives with increasing 4-alkyl chain length, while the number of H-bond acceptors, the number of H-bond donors, and the polar surface area is equal for all tested substances. With increasing alkyl chain length, the number of rotatable bonds and LogP increases, while the aqueous solubility decreases.

### Central and peripheral drug concentrations

Given the observed differences in psychedelic-like effects across the series, despite similar in vitro functional profiles at 5-HT_2A_ receptors, we sought to compare the brain and plasma drug concentrations in mice. We chose to administer drug doses that evoked maximal HTRs as determined from the dose-response experiments depicted in Fig. 2A (1–10 mg/kg). In the single dose experiments, DOM (3 mg/kg), DOPR (1 mg/kg), DOBU (3 mg/kg), DOAM (10 mg/kg), and 2,5-DMA (10 mg/kg) all significantly elevated HTR counts relative to vehicle controls (mean = 19–54 vs. 5), prior to blood and brain collection (Fig. 2G, Table S5). Concentrations of the drugs in plasma were 35–1854 ng/mL (Fig. 2H, Table S6) while whole brain analyses showed 490–6819 ng/g (Fig. 2I, Table S6). All drugs tested were more concentrated in brain tissue vs. plasma, with brain/plasma ratios of 3.9–14.7 (Fig. 2J, Table 3). Brain and plasma drug concentrations were not significantly correlated with HTR counts (data not shown). An interesting observation from the drug concentration data is that longer chain alkyl substitutions yielded lower drug concentrations when compared to those of DOM. For example, DOBU and DOM were given at the same dose, yet the concentrations of DOBU were far less than DOM in both brain and plasma. Similarly, DOAM was administered at a 3-fold higher dose than DOM, and concentrations of DOAM in brain and plasma were similar to those of DOM.

**Table 3:** Brain and plasma levels of 2,5-DMA derivatives after fixed doses inducing maximal HTR in mice.

	Dose	HTR	Plasma levels	Brain tissue levels	Brain/plasma
	[mg/kg]	[count/20 min]	[ng/mL]	[ng/g]	[ratio]
DOM	3	54	397	2459	6.55
DOPR	1	48	35	490	14.67
DOBU	3	37	113	1205	11.73
DOAM	10	30	453	3665	9.63
2,5-DMA	10	19	1854	6819	3.89

**Fig. 3:**
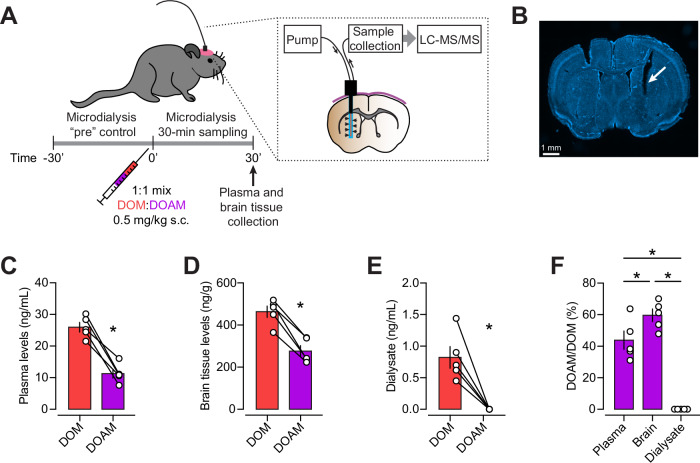
Within-subject microdialysis of DOM and DOAM in the striatum. **A** Experimental details. **B** Representative Hoechst-stained section showing location of microdialysis probe (arrow). DOM and DOAM levels measured in **C** plasma, **D** brain tissue, and **E** dialysate. **F** Levels of DOAM relative to DOM. Data are means ± SEM, *n* = 5 mice. Further statistical information is provided in the results section.

Psychedelics may exhibit high tissue binding in the brain, which reduces the proportion of free, receptor-accessible drug [41]. Additionally, factors such as intracellular sequestration, membrane partitioning, and active transport can contribute to discrepancies between total brain concentrations and effective concentrations at the site of action [60, 61]. This underscores the utility of in vivo microdialysis in estimating unbound drug levels that are more predictive of pharmacodynamic outcomes [62]. Therefore, further studies were conducted to compare brain and plasma drug concentrations, as well as dialysate drug concentrations from cerebral microdialysis, after co-administration of equivalent doses of DOM and DOAM (0.5 mg/kg s.c.; Fig. 3A, B). Both substances had similar in vitro profiles at the 5-HT_2A_ receptor (Table 1), but DOM produced 2.6-fold higher total HTR counts and was more potent in this regard (Fig. 2A, Table 2). Despite being administered at equivalent doses, DOAM was detected at lower levels than DOM in plasma (12 vs. 26 ng/mL, *p* = 0.0017; Fig. 3C) and brain tissue (276 vs. 463 ng/g, *p* = 0.001; Fig. 3D). Mean (SD) recovery of the microdialysis probes was 14.1% (3.5%) for DOM and 3.9% (0.2%) for DOAM. Importantly, DOAM was not detected in dialysate (concentration < 0.04 ng/mL) in contrast to DOM (0.82 ng/mL; Fig. 3E). DOM was therefore more abundant in all matrices, with the biggest difference observed in dialysate (Fig. 3F).

## Discussion

In this study, 4-alkylated 2,5-DMA derivatives bound with highest affinity to 5-HT_2A_ receptors and induced partial to full agonist activities at Gα_q_ and Gα_i/o,12/13_-mediated signaling as well as β-arrestin 2 recruitment at this receptor. Additionally, the substances displayed high affinity and full agonist properties at 5-HT_2B_ and 5-HT_2C_ receptors. Compared to the potent activity at 5-HT_2_ receptors, interactions at other investigated targets were overall mostly minor. Consistent with high activity at 5-HT_2A_ receptors, 2,5-DMA derivatives induced dose-dependent psychedelic-like effects in mice. More specifically, the substances induced the HTR, and compounds with short to intermediate 4-alkyl chain lengths (i.e., DOM and DOPR) were the most potent and efficacious. 2,5-DMA competed for in vitro binding to 5-HT_1A_ and 5-HT_2_ receptors with low micromolar affinity and activated 5-HT_2_ receptors as a full agonist. By comparison, 4-methylation increased the affinity and activity at 5-HT_2_ but not 5-HT_1A_ receptors. Gradually increasing the length of the 4-alkyl chain enhanced binding affinity at 5-HT_2A_ receptors across the series. Similarly, it has previously been shown that agonist activity of serotonergic compounds in sheep umbilical artery strips was enhanced until a three-carbon chain length and then decreased with further extensions [63]. Here, we found that the same rank order applies to all functional assays. The calculated bias factors indicated that some of the compounds were biased towards Gα_q_ or Gα_i/o,12/13_-mediated signaling relative to 5-HT, whereas no β-arrestin 2 bias was observed. Notably, the rank order of potency determined in vitro did not match potencies to induce the HTR, where the compounds with the longest 4-alkyl chains (i.e., DOAM, DOBU) showed reduced potencies and efficacies relative to short or intermediate ones (i.e., DOM, DOPR).

Despite higher affinity of DOBU and DOAM at the 5-HT_2A_ receptor relative to the other compounds tested, these compounds had reduced HTR activity vs. compounds with similar (i.e., DOPR) or reduced affinity (i.e., DOM) at the 5-HT_2A_ receptor. High 5-HT_2A_ receptor affinity with diminished HTR has been reported for 2,5-dimethoxyphenylalkylamines with a bulky 4-thio substituent [22]. Similarly, elongation of the 4-alkyl chain seems to augment antagonistic properties of 2,5-dimethoxyamphetamines by increasing 5-HT_2A_ receptor affinity while reducing receptor activation potency. Clinical potency of psychedelics correlates relatively well with affinity at 5-HT_2A_ and 5-HT_2C_ receptors [42] as well as 5-HT_2A_-mediated β-arrestin 2 recruitment [64]; however, the HTR has also been reported to correlate with G_q_ efficacy but not β-arrestin 2 recruitment at 5-HT_2A_ receptors [26]. Assay specific conditions (i.e., signaling event measured, time of measurement, temperature conditions) may therefore be important for predicting some relationships between the in vitro receptor pharmacology and in vivo behavioral readouts of psychedelics, given the known influence of these factors in cellular assays measuring 5-HT_2A_ functional activities [25, 65]. Moreover, weak 5-HT_2A_ receptor efficacy could lead to a lack of significant psychedelic effects, as observed for various recently described psychedelic analogs [25, 26, 54, 66]. All 2,5-dimethoxyamphetamines assessed in the present study displayed G_q_ efficacies of ≥ 83% and none of the substances was β-arrestin 2-biased. Our study demonstrates that neither 5-HT_2A_ receptor binding nor activation of different G protein-coupled receptor signaling pathways alone is sufficient to completely predict the in vivo potency within a small set of chemically similar psychedelics.

Due to the high structural similarity of the tested substances, ligand docking studies did not reveal any clear SAR trends. Besides the amine group, there are no functional groups that could engage in differential interactions with the binding pocket. It is well established that Asp155 forms a salt bridge with the protonated amine of various psychedelics, including phenethylamines [67–70]. Previous molecular docking studies demonstrated that a hydrophobic region between transmembrane helices IV and V can accommodate bulky 4-substituents, such as a propylthio group, on psychedelic phenethylamines [71]. This suggests that the same region may serve as a binding pocket for the 4-alkyl substituents of the 2,5-DMA derivatives examined in our study. In addition to this hydrophobic interaction, it is plausible that the aromatic ring system of these compounds engages in π–π stacking with aromatic residues in the binding pocket. Several such residues are implicated in ligand binding, including Trp151, Trp336, and Phe339. Trp151 is proposed to participate in π-interactions with ergoline-based psychedelics, although evidence for its involvement with phenethylamines is lacking [70]. Trp336, by contrast, is essential for ligand binding and receptor activation [68]. The psychedelic phenethylamine 25CN-NBOH engages with Trp336, thereby modulating the transition from the inactive to active receptor state [69, 70]. Phe339 is known to form π-cation interactions with the phenethylamine mescaline [70]; another phenylalanine residue, Phe340, has also been implicated in ligand binding for this class of compounds [72]. Interactions involving the 2,5-dimethoxy groups of phenethylamines remain poorly characterized. A hydrophobic contact between the 3’-methoxy group of mescaline and Leu229 on extracellular loop 2 has been reported, as well as a potential electrostatic interaction with Ser242 on helix V [70]. However, mutation of Ser242 to alanine does not affect DOM binding affinity at the 5-HT_2A_ receptor, suggesting that this interaction is not critical for 4-alkylated phenethylamines [73].

When compared to drug affinity at 5-HT_2A_ receptors, the affinity at the 5-HT_2B_ receptor was similar (< 2-fold difference) for all 4-alkylated derivatives. Each compound activated the 5-HT_2B_ receptor as a full agonist, with slightly (~ 3-fold) increased activation potency observed for DOET and DOPR compared to DOM, DOBU, and DOAM. All compounds activated the 5-HT_2C_ receptor as full agonists. Activation potency was highest for derivatives with a two-carbon chain or longer. However, no clear pattern between chain length and activation was evident. Compared to 5-HT_2C_ receptor affinity, activation potencies were around two orders of magnitude higher, which may, at least in part, be explained by differences in the used cell lines. Overall, the 5-HT_2A_ vs. 5-HT_2C_ and 5-HT_2B_ receptor selectivity increased with increasing lipophilicity of the 4-substituent. Of all tested 4-alkylated 2,5-dimethoxyamphetamines, DOM least potently interacted with 5-HT_2C_ receptors and induced the highest amount of HTR counts. This is in line with the notion that 5-HT_2C_ receptor activation opposes HTR [28]. Still, the fact that 5-HT_2C_ receptor binding and activation of DOET and DOAM were similar in the present study, suggests that the decreased potencies and efficacies of derivatives with longer 4-alkyl chain is not solely explained by differential 5-HT_2C_ receptor interactions. In contrast to the 5-HT_2_ receptor subtypes, 4-alkylation did not substantially affect 5-HT_1A_ binding. This finding is consistent with previous studies reporting a higher 5-HT_2A_ vs. 5-HT_1A_ selectivity for psychedelic phenethylamines compared to tryptamines or LSD [2, 9, 15, 74–78]. Differential affinities at 5-HT_1A_ receptors may account for some of the qualitative differences between phenethylamine and tryptamine psychedelics.

Given that little is known about the pharmacological interactions of 4-alkylated 2,5-dimethoxyamphetamines with non-5-HT receptors, we examined interactions at other monoaminergic targets and TAAR1. Compared to the potent interactions with serotonin 5-HT_2_ receptors, the affinities at most other monoaminergic targets were rather weak (i.e., > 1 µM). Similarly, DOPR, DOBU, and DOAM activated TAAR1 at high concentrations only (EC_50_ = 10–22 µM), whereas the other substances did not activate the receptor at the investigated concentration range (EC_50_ > 30 µM). TAAR1 activation has been demonstrated to mediate anti-psychotic and cognition-enhancing activities of other compounds in vivo [79]. Furthermore, TAAR1 antagonism blocks the inhibitory effect of LSD on dopaminergic neurons in the ventral tegmental area [80] and reduces 5-hydroxytryptophan but not psilocybin-induced HTR in mice [81]. Still, there is currently no evidence that weak TAAR1 activity, as in case of DOPR, DOBU, and DOAM, contributes to functional differences in vivo.

DOM and DOPR induced the highest number of twitches (119 and 98, respectively), which gradually decreased with increasing length of the alkyl chain. The HTR induced by DOET was not examined in this study; however, Halberstadt and colleagues previously reported an ED_50_ = 0.20 mg/kg and a maximum of ~122 twitches for DOET [20]. Results from the aforementioned study found the potency and maximal HTR count for DOM to be similar to what we observed here, despite differences in the salt form of the drug used (freebase presently vs. hydrochloride salt) and routes of administration (s.c. presently vs. i.p.). Further, the potency for DOBU (ED_50_ = 1.17 mg/kg) from the Halberstadt et al. study [20] agrees with the present results for the drug (ED_50_ = 1.29 mg/kg) despite differences in route of administration.

Pharmacokinetic factors (i.e., absorption, distribution, metabolism, elimination) may influence the in vivo potency of a psychedelic substance [82, 83]. The summed HTR findings suggest that there may be pharmacokinetic differences across compounds with longer 4-alkyl chain lengths and therefore higher lipophilicity. Importantly, we found that brain and plasma drug concentrations varied after administration of HTR-active doses of the compounds. As an example, we found lower brain concentrations of DOBU vs. DOM when administered at the same 3 mg/kg dose, suggesting a higher volume of distribution of the more lipophilic DOBU or potentially a faster clearance. Notably, 10 mg/kg of DOAM was required to reach brain and plasma concentrations comparable to DOM given at 3 mg/kg. At equal doses, the brain vs. plasma ratio was increased for more lipophilic substances (DOBU vs. DOM, DOAM vs. 2,5-DMA); this aligns with the understanding that lipophilic compounds more readily accumulate in the brain. Nevertheless, compounds with LogP values in the range of 1.5–2.7 are found to display optimal blood–brain barrier penetration [84], whereas excessively high lipophilicity may reduce the amount of drug reaching the receptor site. 2,5-DMA has a LogP value of 1.7 when computed by XLogP3 [85]. 4-Alkylation increases the LogP value as follows: 2.2 (DOM), 2.8 (DOET), 3.4 (DOPR), 4.0 (DOBU), and 4.4 (DOAM). Elongation of the 4-alkyl chain furthermore increases the number of rotatable bonds from four in DOM to eight in DOAM and a high number of rotatable bonds is associated with decreased oral bioavailability [86, 87]. Furthermore, centrally acting substances have generally fewer rotatable bonds, with most CNS drugs having five or fewer rotatable bonds [88]. Compared to their α-desmethyl analogs, 4-substituted 2,5-DMA analogs are metabolically more stable, resulting in long durations of action in vivo [46, 48]. HTR data support this notion, showing stable psychedelic-like effects across the testing session at maximally effective doses. Regardless of stable HTR rate across the test sessions, the drug levels in brain tissue homogenate were not related to HTR activity. This suggests that other factors, such as free drug concentrations at the receptor site, play a more prominent role in pharmacodynamic differences observed for psychedelic-like effects.

To further investigate pharmacokinetic parameters relevant to receptor site exposure, we conducted a microdialysis experiment following the combined systemic administration of low doses of DOM and DOAM. The substances were co-administered at equal doses (0.5 mg/kg each) to minimize inter-animal variability and allow for a direct comparison of extracellular brain concentrations under identical physiological conditions. Consistent with the plasma and brain bioanalysis at peak HTR doses, matched 0.5 mg/kg doses produced higher levels of DOM vs. DOAM. Strikingly, the DOAM concentration in dialysate was below the detection limit of 0.04 ng/mL, indicating that dialysate differences are even more substantial than the differences observed in plasma and brain tissue. It is important to note that the microdialysis experiments were conducted using doses below those required to elicit peak HTR counts; nevertheless, the findings indicate that, as with plasma and brain tissue, higher DOAM doses are required to achieve dialysate concentrations comparable to those of DOM. The observed disconnect between in vitro and in vivo findings demonstrates that early pharmacological screening of new psychedelic drug candidates for therapeutic use should not rely solely on in vitro target and off-target interactions, but should include thorough in vivo pharmacokinetic and pharmacodynamic assessments to determine whether the drugs reach their molecular targets.

## Conclusion

Combining data from studies in humans and animals reveals that 2,5-dimethoxyamphetamines substituted at the 4-position with a short or intermediate unbranched alkyl (i.e., methyl, ethyl, or propyl) chain are potent psychedelics. The increase in potency for 4-alkylated derivatives compared to 2,5-DMA, which lacks a 4-substituent, is a result of substantially increased affinity and activity at the 5-HT_2A_ receptor. However, elongation of the 4-alkyl chain to a butyl or amyl moiety reduces the potency of the respective derivatives in vivo. The observed decrease in psychedelic-like activity of the compounds with the longest 4-alkyl chain lengths does not seem related to reduced activity or efficacy at 5-HT_2A_ receptors or altered interactions with other molecular targets. Here, we show that compounds with the longest 4-alkyl chains required higher doses to reach comparable plasma, brain tissue, and brain dialysate levels. Overall, we propose that elongation of the 4-alkyl chain beyond ethyl or propyl reduces psychedelic effects due to pharmacokinetic factors, including poor blood–brain barrier penetration and reduced central activity, compared to short-chain analogs.

## Supplementary information

Supplementary information

## Data Availability

All data supporting the findings of this study are included in the article and its Supplementary information file. Additional data are available from the corresponding author upon reasonable request.
