# Supplementary material for: The 4-alkyl chain length of 2,5-dimethoxyamphetamines differentially affects in vitro serotonin receptor actions versus in vivo psychedelic-like effects
Source: Mol Psychiatry. 2025 Nov 5;31(3):1799–809. doi: 10.1038/s41380-025-03325-1 (PMC12916490; doi:10.1038/s41380-025-03325-1)
Supplement: Supplementary file 1 — Supplementary information [file 41380_2025_3325_MOESM1_ESM.pdf]

Supplementary information for

The 4-alkyl chain length of 2,5-dimethoxyamphetamines differentially affects *in vitro* serotonin receptor actions versus *in vivo* psychedelic-like effects

Dino Luethi, PhD,[1, #, *] Grant C. Glatfelter, PhD,[2, #] Eline Pottie, PhD,[3] Francesca Sellitti, MS,[4] Alexander D. Maitland, BS,[2] Nicholas R. Gonzalez, BS,[2] Lindsay A. Kryszak, MS,[5] Shelley N. Jackson, PhD,[5] Marius C. Hoener, PhD,[6] Christophe P. Stove, PhD,[3] Matthias E. Liechti, MD,[1] Martin Smieško, PhD,[7] Michael H. Baumann, PhD,[2] Linda D. Simmler, PhD,[4] Deborah Rudin, PhD[1]

[1]Psychopharmacology Research, Department of Biomedicine, University Hospital Basel and University of Basel, Basel, Switzerland
[2]Designer Drug Research Unit, National Institute on Drug Abuse Intramural Research Program, Baltimore, Maryland, United States
[3]Laboratory of Toxicology, Department of Bioanalysis, Faculty of Pharmaceutical Sciences, Ghent University, Ghent, Belgium
[4]Neuropharmacology, Department of Pharmaceutical Sciences, University of Basel, Basel, Switzerland
[5]Translational Analytical Core, National Institute on Drug Abuse Intramural Research Program, Baltimore, Maryland, United States
[6]Neuroscience Research, pRED, Roche Innovation Center Basel, F. Hoffmann-La Roche Ltd, Basel, Switzerland
[7]Computational Pharmacy, Department of Pharmaceutical Sciences, University of Basel, Basel, Switzerland
[#]equal contribution

*Corresponding author: Dr. Dino Luethi, University Hospital Basel, Psychopharmacology Research, ZLF 411, Hebelstrasse 20, 4031 Basel, Switzerland. E-mail: dino.luethi@unibas.ch

This PDF file includes:

1. Supplementary figures (Figure S1–5)
2. Supplementary tables (Table S1–6)
3. Supplementary methods

1. Supplementary figures

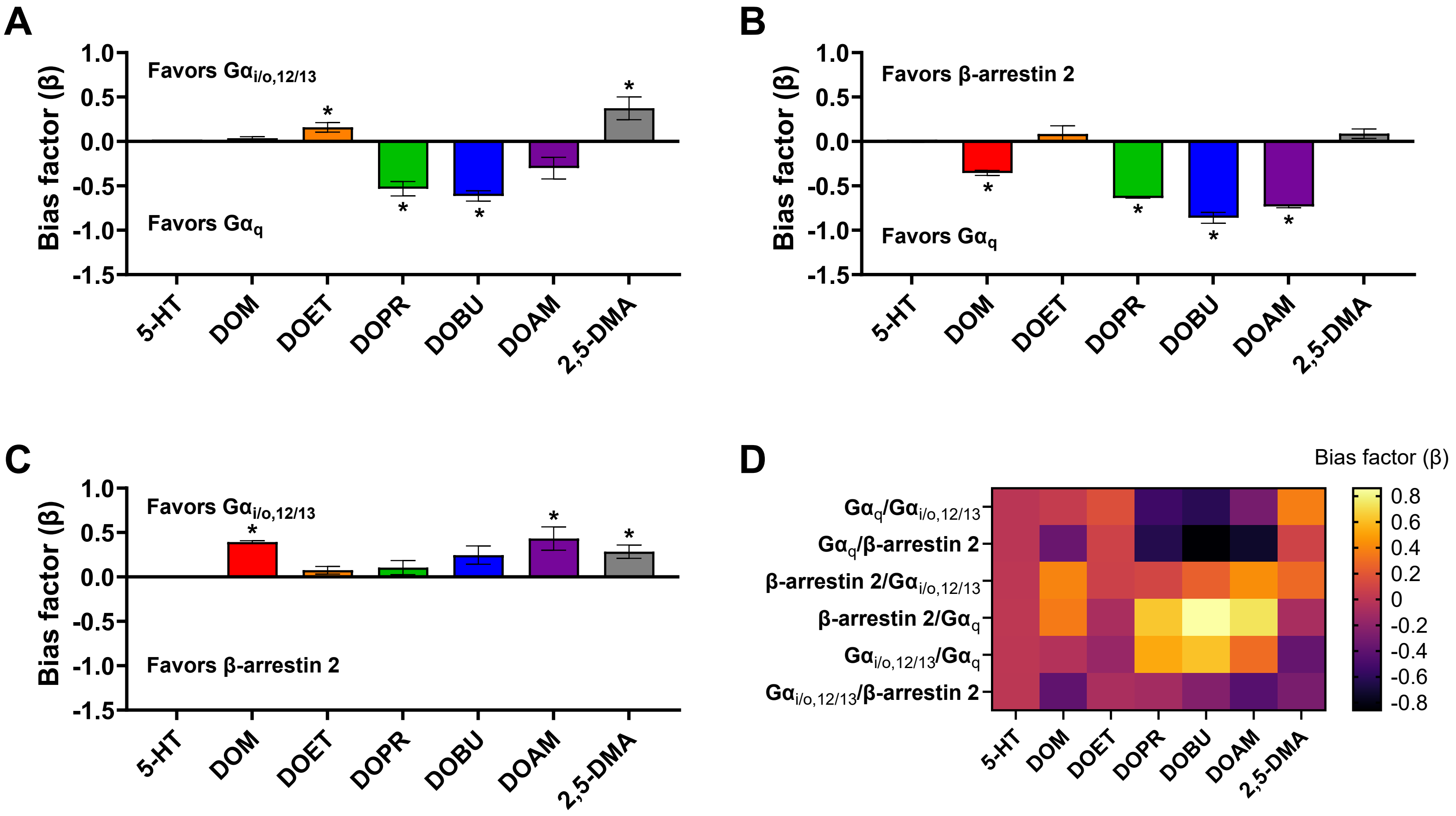

Figure S1. 5-HT_{2A} receptor activation bias, as assessed by the bias factor β. A) Bias toward $G\alpha_{i/o,12/13}$-mediated signaling over $G\alpha_q$-mediated signaling. B) Bias toward β-arrestin 2 recruitment over $G\alpha_q$-mediated signaling. C) Bias toward $G\alpha_{i/o,12/13}$-mediated signaling over β-arrestin 2 recruitment. D) Overview of 5-HT_{2A} receptor activation bias factors**.**

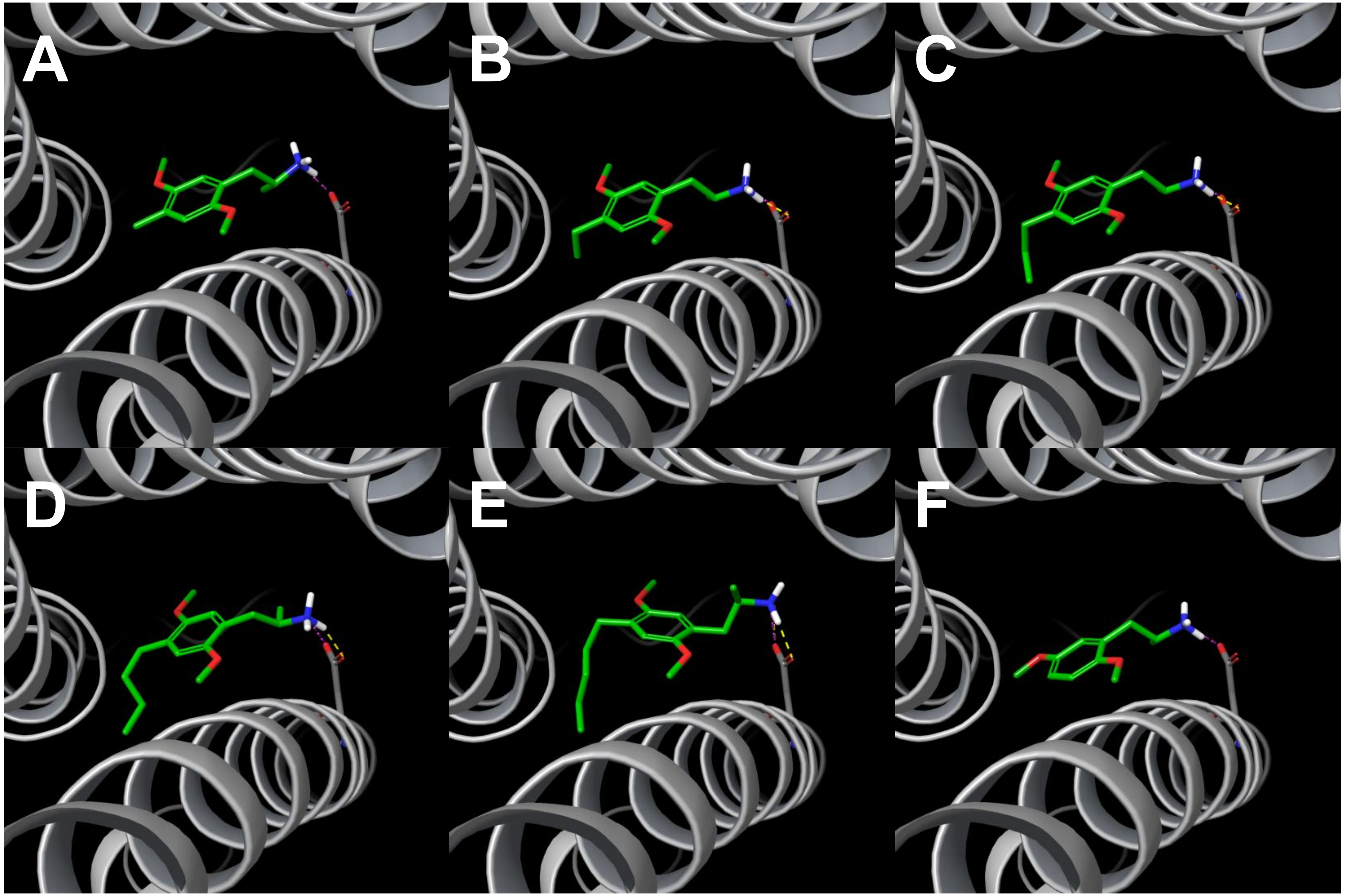

Figure S2. Docked poses and corresponding scores of the (R)-isomers of ligands to the 5-HT_{2A} receptor (PDB ID: 9AS8). A) DOM (-7.8); B) DOET (-7.6); C) DOPR (-7.7); D) DOBU (-7.0); E) DOAM (-7.5); F) 2,5-DMA (-7.4).

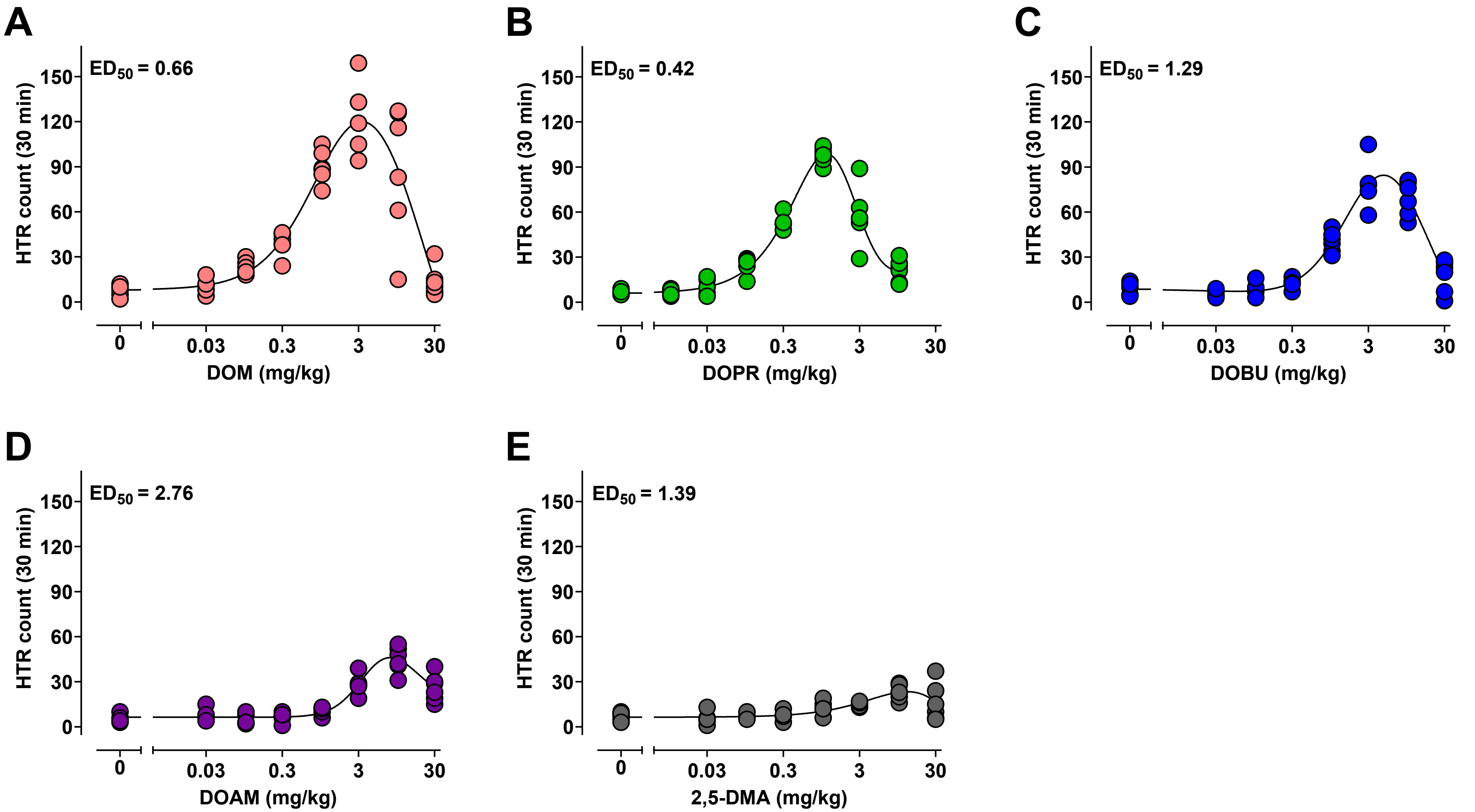

Figure S3. Psychedelic-like effects of 2,5-DMA derivatives varying in 4-alkyl chain length. Dose-response for total number of HTRs induced by A) DOM, B) DOPR, C) DOBU, D) DOAM, and E) 2,5-DMA. Values shown are all mean ± SEM for n = 4–6 mice per dose; *$p < 0.05$. Further statistical information is provided in Table S3.

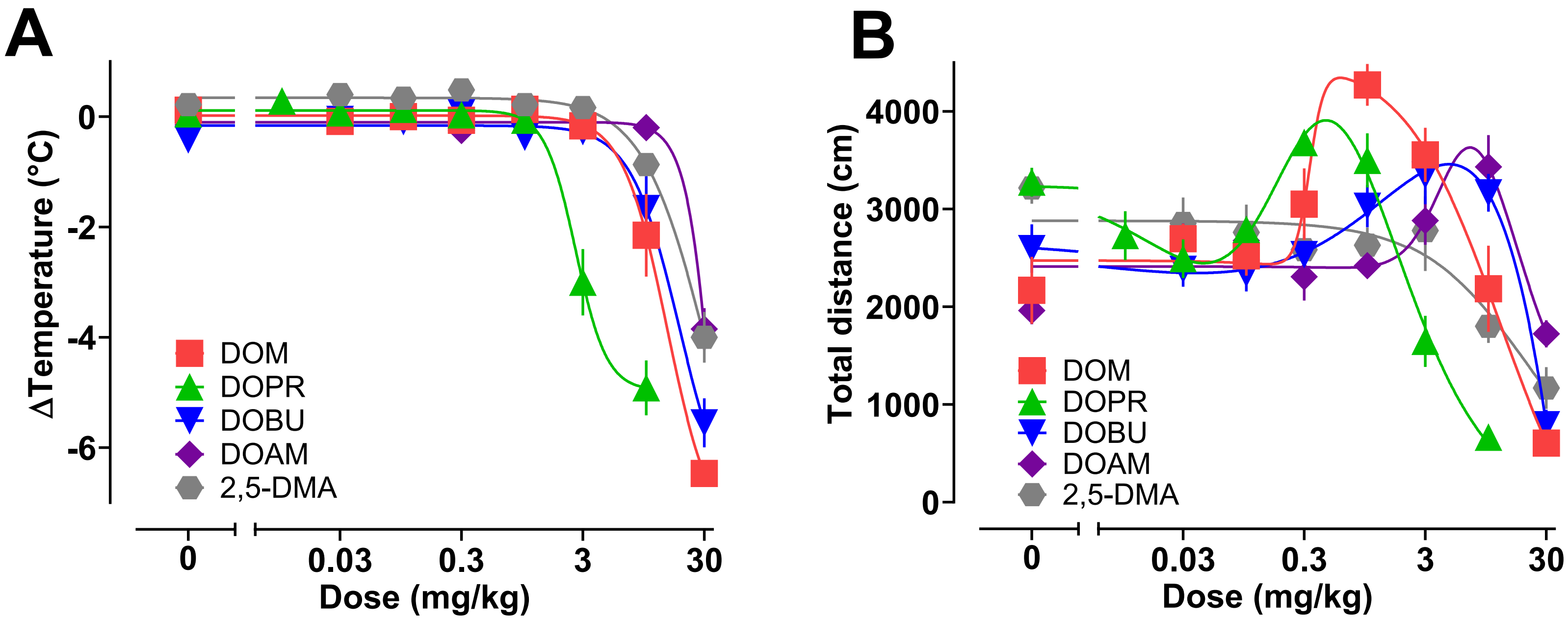

Figure S4. Dose-related effects of 2,5-DMA and its 4-alkylated derivatives on mean body temperature (A) and locomotor activity (B). Values shown are all mean ± SEM for n = 4–6 mice per dose; $*p < 0.05$. Further statistical information is provided in Table S3.

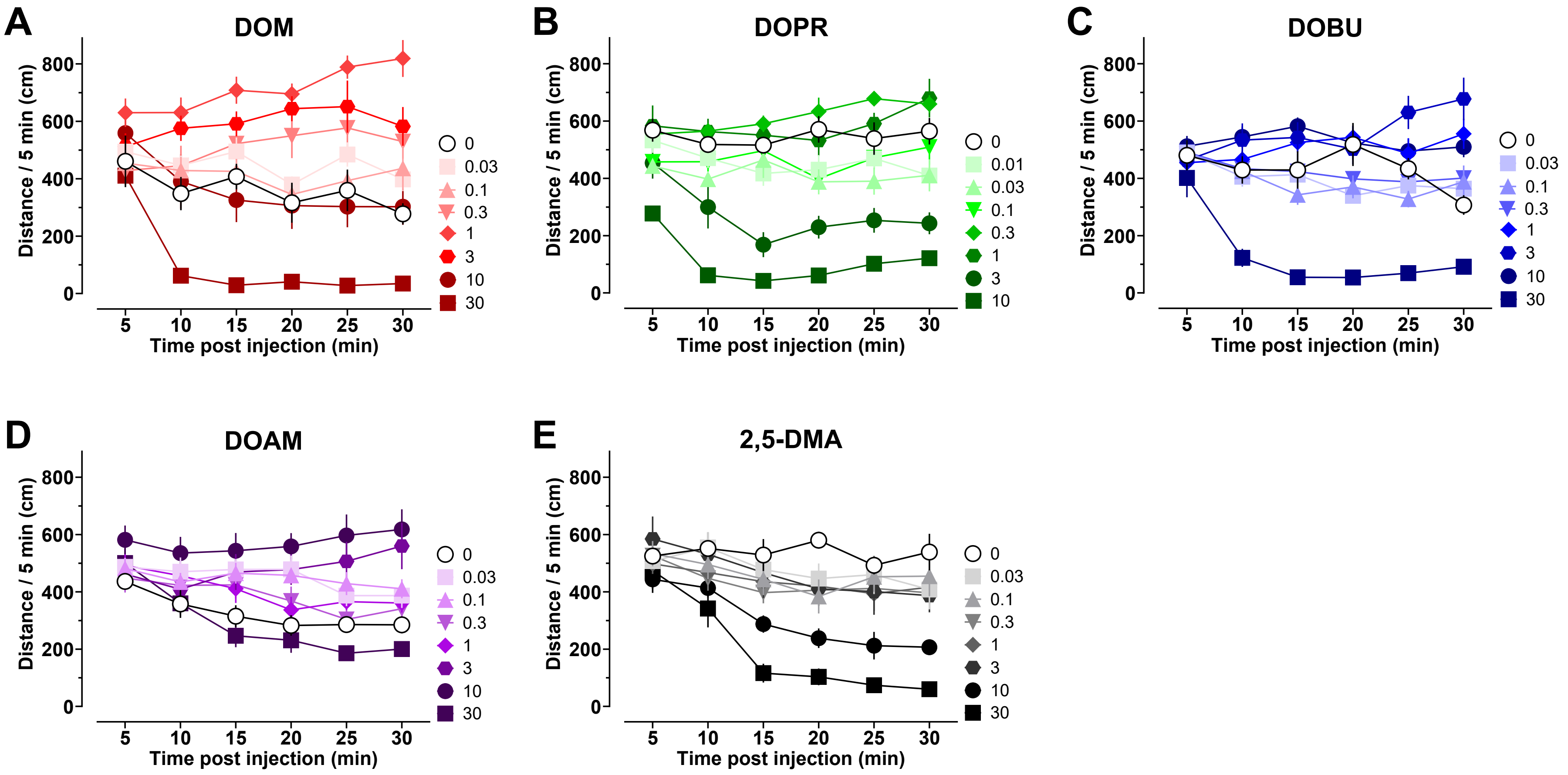

Figure S5. Time-course for effects of 2,5-DMA and its 4-alkylated derivatives on locomotor activity. Locomotion across the testing session for A) DOM, B) DOPR, C) DOBU, D) DOAM, and E) 2,5-DMA. Values shown are all mean ± SEM for n = 4–6 mice per dose. Statistical information is provided in Table S3.

2. Supplementary tables

Table S1. Monoamine receptor and transporter binding affinities.

	hα$_{1A}$	hα$_{2A}$	hD$_2$	hNET	hDAT	hSERT
	K_i [nM]	K_i [nM]	K_i [nM]	K_i [nM]	K_i [nM]	K_i [nM]
DOM	7,393 (7,360–7,426)	> 4,970	> 7,500	> 8,000	> 8,000	> 7,000
DOET	4,006 (3,790–4,222)	> 4,970	> 13,000	> 8,000	> 8,000	> 7,000
DOPR	6,988 (5,921–8,055)	513 (458–568)		> 8,000	> 8,000	> 7,000
DOBU	2,098 (1,900–2,296)	1,714 (1,485–1,943)	> 13,000	> 8,000	> 8,000	> 7,000
DOAM	2,519 (1,985–3,053)	2,308 (2,255–2,361)	> 13,000	> 8,000	3,640 (3,417–3,863)	4,633 (4,536–4,733)
2,5-DMA	5,363 (5,125–5,601)	4,385 (4,082–4,688)	> 13,000	> 8,000	> 8,000	> 7,000

Values are given as mean (95% CI) of at least three experiments.

Table S2. Interactions with TAAR1.

	hTAAR1	
	Activation potency EC_{50} [nM]	Activation efficacy E_{max} [%]
DOM	> 30,000	
DOET	> 30,000	
DOPR	10,148 (5,759–14,536)	95 (85–105)
DOBU	10,427 (5,422–15,432)	98 (91–105)
DOAM	21,848 (18,317–25,379)	86 (65–108)
2,5-DMA	> 30,000	

Values are given as mean (95% CI) of at least three experiments.

Table S3. Mean ± SEM (n = 4–6) and post hoc test comparisons for dose-response effects of DOM and analogues to produce HTR (count/30 min), hypothermia (Δ°C), and hypolocomotion (distance traveled in cm) in mice. Extension of Figure 2. Bold values are statistically significant values vs. saline vehicle control (0 mg/kg). Other relevant statistical information can be found in the materials and methods section. Overall *F*-test values for HTR, temperature change, and locomotor activity comparisons respectively were: DOM ($W_{7.000,16.35}$ = 57.80 p < 0.0001, $W_{7.000, 16.99}$ = 110.3 p < 0.0001, and $W_{7.000,15.84}$ = 69.40 p < 0.0001), DOPR ($W_{7.000,14.47}$ = 199.2 p < 0.0001, $W_{7.000, 16.62}$ = 14.62 p < 0.0001, and $W_{7.000,15.82}$ = 76.25 p < 0.0001), DOBU ($W_{7.000,16.78}$ = 46.83 p < 0.0001, $W_{7.000, 16.92}$ = 18.32 p < 0.0001, and $W_{7.000,16.43}$ = 17.08 p < 0.0001), DOAM ($W_{7.000,16.46}$ = 23.02 p < 0.0001, $W_{7.000,16.89}$ = 11.90 p < 0.0001, and $W_{7.000,17.00}$ = 6.671 p = 0.0007), and 2,5-DMA ($W_{7.000,16.19}$ = 17.82 p < 0.0001, $W_{7.000,17.00}$ = 14.57 p = 0.0001, and $W_{7.000,17.06}$ = 9.802 p = 0.0001).

Drug	Dose [mg/kg] (*n*)	HTR [count]	Post test *p* value	ΔTemperature [°C]	Post test *p* value	Locomotor activity [cm]	Post test *p* value
DOM	0 (6)	7.5 ± 1.5	–	0.1 ± 0.2	–	2,170 ± 345	–
	0.03 (6)	10.2 ± 1.9	0.8724	-0.1 ± 0.1	0.9656	2,696 ± 154	0.7128
	0.1 (5)	**23.4 ± 2.1**	**0.0028**	0.0 ± 0.1	0.9976	2,522 ± 203	0.9492
	0.3 (6)	**39.0 ± 3.3**	**0.0003**	-0.1 ± 0.1	0.9839	3,051 ± 367	0.4932
	1 (6)	**90.0 ± 4.4**	**< 0.0001**	0.1 ± 0.1	> 0.9999	**4,272 ± 215**	**0.0052**
	3 (6)	**119.2 ± 9.7**	**0.0005**	-0.2 ± 0.1	0.8533	3,556 ± 278	0.0635
	10 (6)	**88.0 ± 18.1**	**0.0350**	-2.2 ± 0.8	0.1391	2,186 ± 443	> 0.9999
	30 (6)	13.7 ± 3.9	0.6771	**-6.5 ± 0.2**	**< 0.0001**	**606 ± 47**	**0.0330**
DOPR	0 (6)	7.2 ± 0.7	–	0.1 ± 0.1	–	3,275 ± 149	–
	0.01 (6)	6.2 ± 0.9	0.9409	0.3 ± 0.2	0.8957	2,728 ± 251	0.4344
	0.03 (6)	9.7 ± 2.2	0.8648	0.1 ± 0.1	> 0.9999	2,493 ± 199	0.0685
	0.1 (6)	**22.7 ± 2.8**	**0.0099**	0.1 ± 0.2	0.9997	2,791 ± 131	0.1969
	0.3 (4)	**53.6 ± 2.9**	**0.0025**	0.0 ± 0.2	> 0.9999	3,680 ± 100	0.2589
	1 (6)	**98.2 ± 2.3**	**< 0.0001**	-0.1 ± 0.1	0.9791	3,500 ± 278	0.9803
	3 (5)	**58.0 ± 9.6**	**0.0294**	**-3.0 ± 0.6**	**0.0150**	**1,648 ± 262**	**0.0040**
	10 (6)	**21.2 ± 3.0**	**0.0332**	**-4.9 ± 0.5**	**0.0004**	**665 ± 82**	**< 0.0001**

Table S3 (continued).

DOBU	0 (6)	9.2 ± 1.6	–	-0.4 ± 0.2	–	2,597 ± 247	–
	0.03 (6)	6.0 ± 0.9	0.5350	-0.1 ± 0.1	0.3903	2,386 ± 181	0.9860
	0.1 (6)	9.0 ± 1.7	> 0.9999	-0.1 ± 0.2	0.7032	2,342 ± 184	0.9640
	0.3 (6)	12.7 ± 1.3	0.5353	0.1 ± 0.1	0.2456	2,535 ± 61	> 0.9999
	1 (6)	**40.2 ± 2.9**	**< 0.0001**	-0.4 ± 0.1	> 0.9999	3,030 ± 217	0.7591
	3 (6)	**78.7 ± 6.2**	**0.0002**	-0.3 ± 0.2	0.9912	3,348 ± 297	0.3879
	10 (6)	**69.3 ± 4.7**	**0.0001**	-1.7 ± 0.6	0.3730	1,167 ± 195	0.4645
	30 (6)	17.5 ± 4.5	0.5160	**-5.5 ± 0.4**	**0.0002**	**793 ± 154**	**0.0016**
DOAM	0 (6)	5.3 ± 1.0	–	-0.2 ± 0.1	–	1,961 ± 142	–
	0.03 (6)	7.8 ± 1.6	0.7444	0.0 ± 0.1	0.7497	**2,867 ± 167**	**0.0473**
	0.1 (5)	6.2 ± 1.6	0.9984	0.0 ± 0.1	0.8949	**2,677 ± 103**	**0.0170**
	0.3 (6)	6.5 ± 1.2	0.9816	-0.3 ± 0.2	0.9979	2,307 ± 244	0.8088
	1 (6)	9.5 ± 1.2	0.1398	-0.1 ± 0.2	0.9995	2,421 ± 129	0.2032
	3 (6)	**28.5 ± 2.6**	**0.0004**	-0.2 ± 0.1	> 0.9999	**2,884 ± 249**	**0.0698**
	10 (6)	**44.8 ± 3.6**	**0.0002**	-0.2 ± 0.2	> 0.9999	**3,433 ± 325**	**0.0248**
	30 (6)	**26.0 ± 3.7**	**0.0090**	**-3.9 ± 0.4**	**0.0005**	1,724 ± 138	0.8239
2,5-DMA	0 (6)	7.0 ± 1.1	–	0.2 ± 0.1	–	3,216 ± 162	–
	0.03 (5)	5.8 ± 1.9	0.9960	0.4 ± 0.1	0.8691	2,854 ± 266	0.8408
	0.1 (6)	6.7 ± 0.7	> 0.9999	0.3 ± 0.1	0.9906	2,763 ± 284	0.7180
	0.3 (6)	7.3 ± 1.2	> 0.9999	0.5 ± 0.1	0.6610	2,584 ± 157	0.1075
	1 (6)	11.7 ± 2.1	0.3842	0.2 ± 0.1	> 0.9999	2,630 ± 184	0.2032
	3 (6)	**15.3 ± 0.6**	**0.0009**	0.2 ± 0.2	> 0.9999	2,780 ± 415	0.9149
	10 (6)	**23.2 ± 1.9**	**0.0006**	**-0.9 ± 0.2**	**0.0090**	**1,801 ± 171**	**0.0009**
	30 (6)	16.2 ± 5.0	0.5132	**-4.0 ± 0.5**	**0.0007**	**1,169 ± 214**	**0.0002**

Table S4. Blood-brain penetration parameters predicted with the QikProp tool.

	DOM	DOET	DOPR	DOBU	DOAM	2,5-DMA
CIQPlogS	-1.672	-1.944	-2.219	-2.496	-2.776	-1.404
QPlogBB	0.288	0.221	0.148	0.075	-0.151	0.303
#Rotatable bonds	4	5	6	7	8	4
#H-bond acceptors	3	3	3	3	3	3
#H-bond donors	1	1	1	1	1	1
TPSA (Å^2)	44.48	44.48	44.48	44.48	44.48	44.48
XLogP3	2.24	2.81	3.37	4.00	4.43	1.72

CIQPlogS: conformation-independent predicted aqueous solubility; QPlogBB: predicted brain/blood partition coefficient; TPSA: topological polar surface area.

Table S5. Mean ± SEM (n = 9–10) and post hoc test comparisons for effects of DOM and analogs to produce HTR (count/20 min) in mice prior to brain and blood collection for PK analyses. Extension of Figure 2. Bold values are statistically significant values vs. saline vehicle control (0 mg/kg). Other relevant statistical information can be found in the materials and methods section. The overall Welch's ANOVA value for these comparisons was $W_{5.000,22.70} = 75.15$ $p < 0.0001$.

Drug	Dose [mg/kg] (*n*)	HTR count/20 min [mean ± SEM]	Post test *p* value
Vehicle	0 (10)	4.5 ± 0.7	–
DOM	3 (10)	**53.4 ± 5.9**	**< 0.0001**
DOPR	1 (9)	**47.7 ± 5.4**	**0.0002**
DOBU	3 (10)	**37.3 ± 2.8**	**< 0.0001**
DOAM	10 (10)	**29.6 ± 1.5**	**< 0.0001**
2,5-DMA	10 (10)	**18.9 ± 2.0**	**0.0002**

Table S6. Mean (n = 9–10) and post hoc test comparisons for brain tissue and plasma levels of HTR maximal doses of 2,5-DMA derivatives in mice. Extension of Figure 2. Bold values are statistically significant values vs. DOM. Other relevant statistical information can be found in the materials and methods section. The overall Welch's ANOVA values for measures included were: plasma levels ($W_{4.000,19.23}$ = 59.68 p < 0.0001), brain levels ($W_{4.000,19.98}$ = 137.4 p < 0.0001), and brain/plasma ratio ($W_{4.000,19.56}$ = 19.35 p < 0.0001).

Drug	Dose [mg/kg] (*n*)	Plasma levels [ng/mL] (95% CI)	Post test *p* value	Brain levels [ng/g] (95% CI)	Post test *p* value	Brain/plasma [ratio] (95% CI)	Post test *p* value
DOM	3 (10)	397 (314–480)	–	2,459 (2,113–2,805)	–	6.55 (5.32–7.78)	–
DOPR	1 (9)	**35** (25–44)	**< 0.0001**	**490** (407–572)	**< 0.0001**	**14.67** (10.62–18.71)	**0.0049**
DOBU	3 (10)	**113** (89–137)	**< 0.0001**	**1,205** (1,047–1,363)	**< 0.0001**	**11.73** (8.48–14.98)	**0.0210**
DOAM	10 (10)	453 (308–599)	0.9031	**3,665** (3,000–4,329)	**0.0103**	9.63 (6.25–13.01)	0.2592
2,5-DMA	10 (10)	**1,854** (1,421–2,287)	**< 0.0001**	**6,819** (6,000–7,637)	**< 0.0001**	**3.89** (3.24–4.55)	**0.0027**

3. Supplementary methods

3.1 Assessment of receptor and transporter binding affinities

Membrane preparations were prepared from cells transfected with the respective target. The membrane preparations were then incubated with selective radioligands at concentrations equal to the corresponding K_d and displacement of the ligand by the drugs was measured. The difference between total binding and nonspecific binding (determined in the presence of selective competitors) was defined as specific binding of the radioligand to the target receptor. The following transfected cell lines were used for the binding assays: HEK 293 cells (h5-HT_{1A}, h5-HT_{2A}, h5-HT_{2B}, h5-HT_{2C}, and hD_2 receptors, hDAT, hNET, and hSERT), Chinese hamster ovary cells ($h\alpha_{1A}$ receptor), and Chinese hamster lung cells ($h\alpha_{2A}$ receptor).

3.1.1 Membrane preparations for receptor and transporter binding

For producing membrane preparations, transfected cells were harvested with trypsin/EDTA, washed with ice-cold phosphate buffered saline, and then pelleted by centrifugation (210 × g for 5 min at 4 °C). The pellets were frozen and stored at -80 °C until use. Frozen pellets were resuspended in 20 mL (receptor binding) or 400 mL (transporter binding) buffer (20 mM HEPES-NaOH, 10 mM EDTA, pH 7.4), and homogenized at 1,100 × g for 20 s (receptor binding) or 560 × g for 15 s (transporter binding); the homogenates were then centrifuged at 48,000 × g for 30 min at 4 °C. For receptor binding assays, the supernatants were discarded and the pellets were resuspended in 20 mL buffer (20 mM HEPES-NaOH, 0.1 mM EDTA, pH 7.4) and homogenized at 1,100 × g for 20 s. Centrifugation and discarding of the supernatant were repeated once, and the final pellet was then resuspended in buffer and homogenized.

3.1.2 Serotonin 5-HT_{1A} and 5-HT_{2A} receptor binding

Competitive binding at the h5-HT_{1A} and h5-HT_{2A} receptor was determined using 0.90 nM [^{3}H]8-hydroxy-2-(di-*n*-propylamine)tetralin ([^{3}H]8-OH-DPAT; h5-HT_{1A} receptor agonist) and 0.40 nM [^{3}H]ketanserin (h5-HT_2 receptor antagonist) as radioligands. The density and affinity of [^{3}H]8-OH-DPAT binding sites were 2.77 pmol/mg protein and 0.68 nM, respectively. The density and affinity of [^{3}H]ketanserin binding sites were 19.89 pmol/mg protein and 0.37 nM, respectively. Briefly, specific binding of the radioligands was defined as difference between total binding (binding buffer alone) and nonspecific binding determined in the presence of 10 µM pindolol (h5-HT_{1A} receptor antagonist) or 10 µM spiperone (h5-HT_{2A} receptor antagonist). Dilution curves of test compounds in binding assay buffer (50 mM Tris/HCl, 10 mM $MgCl_2$, and 1 mM EGTA, pH 7.4) were prepared in 96-well round bottom polystyrene microplates (Greiner Bio-One, Kremsmünster, Austria). Radioligand (50 µL) and membrane suspension (100 µL) were then added to the assay plates and incubated for 30 min under shaking at room temperature. The incubations were terminated by rapid filtration through UniFilter-96 plates with GF/C glass filters

(Packard Instrument Company, RI, USA) that were presoaked in 0.3% polyethylenimine and washed three times with ice-cold washing buffer (50 mM Tris/HCl, pH 7.4). Afterwards, MicroScint 40 (Perkin Elmer, Schwerzenbach, Switzerland) was added (45 µL/well) and the UniFilter-96 plates were sealed. After 1 h, the radioactivity was measured with a TopCount Microplate Scintillation Counter (Packard Instrument Company).

3.1.3 Serotonin 5-HT$_{2B}$ receptor binding

Competitive binding at the h5-HT$_{2B}$ receptor was determined using 1 nM [^{3}H]LSD (5-HT$_{2B}$ receptor agonist) as radioligand. The density and affinity of [^{3}H]LSD binding sites were 4.49 pmol/mg protein and 3.13 nM, respectively. Specific binding of the radioligand was defined as difference between total binding (binding buffer alone) and nonspecific binding determined in the presence of 100 µM 5-HT. Dilution curves of test compounds in binding assay buffer (50 mM Tris/HCl, 10 mM $MgCl_2$, and 1 mM EGTA, pH 7.4) were prepared in 96-well flat bottom polystyrene microplates (BRAND, Wertheim, Germany). Radioligand (50 µL) and membrane suspension (100 µL) were then added to the assay plates and incubated for 60 min at 37 °C. The incubations were terminated by rapid filtration through UniFilter-96 plates with GF/C glass filters (Packard Instrument Company) that were presoaked in 0.5% polyethylenimine and washed three times with ice-cold washing buffer (50 mM Tris/HCl, pH 7.4). Afterwards, MicroScint 20 (Perkin Elmer) was added (30 µL/well) and the UniFilter-96 plates were sealed. After 24 h, the radioactivity was measured with a TopCount Microplate Scintillation Counter (Packard Instrument Company).

3.1.4 Serotonin 5-HT$_{2C}$ receptor binding

Competitive binding at the h5-HT$_{2C}$ receptor was determined using 1.4 nM [^{3}H]mesulergine (5-HT$_{2C}$ receptor antagonist) as radioligand. The density and affinity of [^{3}H]mesulergine binding sites were 1.36 pmol/mg protein and 1.59 nM, respectively. Specific binding of the radioligand was defined as the difference between total binding (binding buffer alone) and nonspecific binding determined in the presence of 10 µM mianserin (5-HT$_{2C}$ receptor inverse agonist). Dilution curves of test compounds in binding assay buffer (50 mM Tris/HCl, 10 mM $MgCl_2$, 1 mM EGTA, and 10 µM pargyline, pH 7.4) were prepared in white 96-well polystyrene assay plates (Sigma-Aldrich, Buchs, Switzerland). The membrane homogenate (40 µg/mL) was lightly mixed for 5–30 min with YSi-poly-L-lysine (PerkinElmer) at 0.5 mg beads/well and the mixture (50 µL) was then added to each well of the assay plate containing radioligand (50 µL) and test compounds (final volume of 200 µL). The assay plates were sealed and incubated for 2 h at room temperature with agitation. Thereafter, radioactivity was measured in the PVT SPA counting mode of a TopCount Microplate Scintillation Counter (Packard Instrument Company).

3.1.5 Adrenergic α_{1A} and α_{2A} receptor binding

Competitive binding at the hα_{1A} and hα_{2A} receptor was determined using 0.11 nM [^{3}H]prazosin (α_1 receptor antagonist) and 2 nM [^{3}H]rauwolscine (α_2 receptor antagonist) as radioligands. The density and affinity of [^{3}H]prazosin binding sites were 3.01 pmol/mg protein and 0.04 nM, respectively; the density and affinity of [^{3}H]rauwolscine binding sites were 3.83 pmol/mg protein and 2.02 nM, respectively. Specific binding of the radioligands was defined as the difference between total binding (binding buffer alone) and nonspecific binding that was determined in the presence of 10 µM chlorpromazine (α_{1A} receptor antagonist) or 10 µM phentolamine (α_{2A} receptor antagonist). Dilution curves of the test compounds diluted in binding assay buffer (50 mM Tris/HCl, pH 7.4) were prepared in 96-well round bottom polystyrene microplates (Greiner Bio-One). Radioligands (50 µL) and membrane suspension (100 µL) were added to the assay plates and incubated for 1 h under shaking at room temperature. The incubations were terminated by rapid filtration through UniFilter-96 plates with GF/C glass filters (Packard Instrument Company) that were presoaked in 0.3% polyethylenimine and washed three times with 1 mL cold binding assay buffer. Thereafter, MicroScint 40 (45 µL/well, PerkinElmer) was added, the UniFilter-96 plates were sealed, and radioactivity was measured after 1 h using a TopCount Microplate Scintillation Counter (Packard Instrument Company).

3.1.6 Dopamine D_2 receptor binding

Competitive binding at the hD_2 receptor was determined using 1.2 nM [^{3}H]spiperone (D_2 receptor antagonist) as radioligand. The density and affinity of [^{3}H]spiperone binding sites were 18.34 pmol/mg protein and 0.26 nM, respectively. Specific binding of the radioligand was defined as the difference between total binding (binding buffer alone) and nonspecific binding determined in the presence of 10 µM spiperone. Dilution curves of the test compounds diluted in binding assay buffer (50 mM Tris/HCl, 5 mM $MgCl_2$, 1 mM EDTA, 5 mM KCl, 1.5 mM $CaCl_2$, and 120 mM NaCl, pH 7.4) were prepared in 96-well round bottom polystyrene microplates (Greiner Bio-One). Radioligand (50 µL) and the membrane suspension (100 µL) were added to the assay plates and incubated for 1 h at room temperature under shaking. Incubations were terminated by rapid filtration through UniFilter-96 plates with GF/C glass filters (Packard Instrument Company) presoaked in 0.3% polyethylenimine and were then washed three times with ice-cold washing buffer (50 mM Tris/HCl, pH 7.4). MicroScint 40 (45 µL/well, PerkinElmer) was added and the UniFilter-96 plates were sealed. The radioactivity was measured after 1 h using a TopCount Microplate Scintillation Counter (Packard Instrument Company).

3.1.7 Monoamine transporter binding

Competitive binding at human monoamine transporters was determined using 2.9 nM *N*-methyl-[^{3}H]nisoxetine (B_{max}: 0.79 pmol/mg protein, K_d: 10.44 nM), 3.3 nM [^{3}H]WIN35,428 (B_{max}: 10.36 pmol/mg protein, K_d: 16.51 nM), and 1.5 nM [^{3}H]citalopram (B_{max}: 12.04 pmol/mg protein, K_d: 8.85 nM) as radioligands for hNET, hDAT, and hSERT, respectively. Specific binding of the radioligand was defined as the difference between total binding (binding buffer alone) and nonspecific binding that was determined in the presence of 10 μM indatraline. The test compounds were diluted in binding assay buffer at pH 7.4 (126 mM NaCl, 2.7 mM KCl, 10 mM Na_2HPO_4, and 1.76 mM KH_2PO_4), and dilution curves were prepared in 96-well OptiPlates (PerkinElmer). Membrane stocks were resuspended in binding assay buffer (60 μg protein/mL) using a Polytron tissue homogenizer. The membrane homogenates were then gently mixed for 5–30 min with polyvinyl toluene wheat germ agglutinin-coated scintillation proximity assay beads (11.5 mg beads/well; WGA-SPA, Amersham Biosciences, Amersham, UK). Thereafter, the membrane/bead mixture (50 μL) was added to each well of the assay plate that contained the radioligand (50 μL) and the test compounds (final volume of 200 μL). The assay plates were sealed and incubated for 2 h with agitation at room temperature. Radioactivity was then measured in the PVT SPA counting mode of a TopCount Microplate Scintillation Counter (Packard Instrument Company).

3.2 Functional activity at 5-HT receptors

3.2.1 IP_1 formation

Activation of the 5-HT_{2A}, 5-HT_{2B}, and 5-HT_{2C} receptor was assessed by measuring the accumulation of inositol monophosphate (IP_1) as a marker for $G\alpha_q$ mediated signaling, using the Cisbio IP-One Gq Kit (Cisbio Bioassays SAS, Codolet Cedex, France) according to the manufacturer's protocol. In brief, NIH/3T3 cells (CRL-1658, ATCC, Manassas, Virginia, U.S.) stably expressing the human 5-HT_{2A} receptor were seeded at a density of 2,500 cells per well in a 384-well plate in Opti-MEM™ I medium (Gibco, ThermoFisher, Life Technologies Europe B.V., Zug, Switzerland). Stock solutions (10 mM) of test substances were prepared in DMSO and further diluted in assay buffer containing lithium chloride. Test compounds were added to the cells, and the plate was incubated for 90 min at 37 °C, followed by 60 min incubation with Anti-IP_1-Cryptate and IP_1-d_2 at room temperature. Stimulated IP_1 formation was determined by Homogeneous Time-Resolved Fluorescence (HTRF) measurement on a BioTek Synergy H1 Multimode Reader (Agilent Technologies AG, Basel, Switzerland) and EC_{50} values were derived from the concentration-response curves using nonlinear regression. All data were normalized to the maximal effect of 5-HT, which was measured in parallel.

3.2.2 β-arrestin 2 recruitment

HEK 293T cells stably expressing 5-HT_{2A}-LgBiT and SmBiT-β-arrestin 2 (being the receptor and the β-arrestin 2 molecule in the NanoBiT system) were seeded at a density of 50,000 cells per well in poly-D-lysine-coated 96-well plates in medium containing 10% dialyzed FBS. The plate was then incubated at 37 °C and 5% CO_2. The following day, the cells were washed twice with 150 µL HBSS, and 100 µL HBSS was subsequently added to each well. Thereafter, 25 µL of substrate (1:20 diluted in buffer) was added, and the plate was transferred to a TriStar^2 LB 942 multimode microplate reader (Berthold Technologies, Bad Wildbad, Germany). After equilibration of the luminescent signal, 10 µL of the 13.5-fold concentrated agonist solutions was added, and the signal was monitored for 2 h. Drug dilutions in HBSS were made from 10 mM stock solutions in DMSO, yielding concentration ranges from 10 µM to 10 pM. All concentrations were run in duplicate and included the appropriate solvent controls. Efficacy was normalized to 5-HT activity.

3.2.3 Arachidonic acid release

Arachidonic acid release was assessed as a marker for $G\alpha_{i/o,12/13}$ mediated signaling. NIH/3T3 cells stably expressing the 5-HT_{2A} receptor were seeded at a density of 75,000 cells per well in a poly-D-lysine-coated 48-well plate. The following day, the cells were labeled with 300 µL [^{3}H]arachidonic acid (0.3 µCi/mL) in serum-free medium for 4 h at 37 °C. Thereafter, the cells were washed 3 times for 5 min at 37 °C with culture medium containing 2% fatty acid free bovine serum albumin. Stock solutions (10 mM) of test substances were prepared in DMSO. The final dilution series of test substances dissolved in medium (500 µL) was then added for 30 min at 37 °C. Thereafter, the plate was put on ice, and 400 µL incubation supernatant per well was transferred into scintillation vials containing scintillation fluid (Ultimagold, Perkin Elmer, Schwerzenbach, Switzerland). The remaining supernatant was removed, and the cells were lysed with 1% sodium dodecyl sulfate lysis buffer. The obtained cell lysate was transferred into scintillation vials containing scintillation fluid. Radioactivity was measured on a liquid scintillation counter (Packard 1900 TR Tri-Carb Liquid Scintillation Counter; Packard Instrument Company). Efficacy was normalized to 5-HT activity.

3.2.4 Calculation of bias factors

For each 2,5-DMA derivative, relative activity (RA) values for each pathway were calculated using 5-HT as the reference agonist.

$$RA_{i,\,ref\ agonist}^{pathway} = \frac{\frac{E_{max,\,i}}{EC_{50,\,i}}}{\frac{E_{max,\,ref}}{EC_{50,\,ref}}} = \frac{EC_{50,\,ref} \times E_{max,i}}{E_{max,\,ref} \times EC_{50,i}}$$

Then, the obtained RA values were used to calculate the bias factor (β) for each pathway combination.

$$\beta_i = log\left(\frac{RA_{i,ref}^{pathway\ 1}}{RA_{i,ref}^{pathway\ 2}}\right)$$

The reference agonist 5-HT has a β of zero, whereas agonists with a positive β value preferentially induce pathway 1 over pathway 2. Brown-Forsythe and Welch ANOVA analyses were applied to estimate the statistical probability that β is different from zero.

3.3 Functional activity at the human TAAR1

HEK 293 cells (CRL-1573, ATCC) that stably expressed the human trace amine-associated receptor 1 (hTAAR1) were generated by transfection with Lipofectamine 2000 (Invitrogen, Zug, Switzerland). The cells were harvested and pelleted by centrifugation at 900 rpm for 3 min at room temperature. The supernatant was then removed, and the cell pellet was resuspended in fresh culture medium. The cells were plated into 96-well plates (100 µL, containing 80,000 cells per well) and incubated for 20 h at 37 °C. The culture medium was removed, and the cells were washed once with 50 µL phosphate buffered saline without Ca^{2+} and Mg^{2+}. Thereafter, 90 µL of Krebs-Ringer bicarbonate buffer (Sigma-Aldrich) containing 1 mM IBMX was added, and the plates were incubated for 60 min at 37 °C. A broad concentration range (300 pM to 30 µM) of the test compounds was tested, and a standard curve (0.13 nM to 10 µM cAMP) was included on each plate. In addition, a reference plate containing RO5256390, β-phenylethylamine, and *p*-tyramine was included in each experiment. Test compound solution, β-phenylethylamine (as maximal response), or basal control were added at a volume of 30 µL, and the cells were incubated for 40 min at 37 °C. Afterwards, the cells were lysed with 50 µL of detection mix solution containing Ru-cAMP Alexa700 anti-cAMP antibody and lysis buffer for 120 min at room temperature under heavy shaking. Fluorescence was then measured.

3.4 Plasma and brain tissue analysis

3.4.1 Analytical standards

Stock solutions of DOM, 2,5-DMA, DOPR, DOBU, and DOAM were diluted in DMSO and used to spike blank plasma or tissue homogenate at calibration standards of 1, 2.5, 5, 10, 25, 50, 100, 250, and 500 ng/mL.

3.4.2 Plasma analysis

Extraction was performed by protein precipitation with 150 µL of acetonitrile added to 50 µL plasma in a 2-mL centrifuge tube. The samples were then vortexed for 10 s and shaken for 3 min at

2,000 rpm with a ThermoMixer C (Eppendorf, Enfield, CT). The samples were centrifuged at 10,000 g for 10 min at 4 °C in a Sorvall ST 40R centrifuge (ThermoFisher, Waltham, MA). Supernatant (150 µL) was collected in a LC glass vial and evaporated to dryness at 50 °C under a stream of nitrogen gas in a nitrogen evaporator. The residue was resuspended with 50 µL of 50% methanol in 0.1% formic acid and shaken at 1,000 rpm for 5 min. Samples were transferred to the autosampler where 10 µL was injected into the UHPLC–MS system.

3.4.3 Brain tissue analysis

Ultrapure water was added at 4 µL per mg of tissue and tissue was homogenized using a Bead Ruptor Elite homogenizer (Omni International, Kennesaw, GA). Extraction was performed by protein precipitation with 400 µL of acetonitrile to 100 µL tissue homogenate in a 2-mL centrifuge tube. The samples were then vortexed for 10 s and shaken for 3 min at 1,000 rpm with a ThermoMixer C. The samples were centrifuged at 10,000 g for 10 min in a Sorvall ST 40R centrifuge. Supernatant (400 µL) was collected in a 0.5-mL skirted centrifuge tube and evaporated to dryness at 50 °C under a stream of nitrogen gas in a nitrogen evaporator. The residue was resuspended with 100 µL of 50% methanol in 0.1% formic acid, shaken at 1,000 rpm for 5 min, and transferred to a 0.5-mL centrifuge tube. Next, samples were centrifuged at 10,000 g for 10 min at 4 °C and 80 µL of the supernatant was collected and placed in a LC vial and transferred to the autosampler where 10 µL was injected into the UHPLC-MS system.

3.4.4 LC–MS

Sample analysis was performed using a Vanquish UHPLC system (ThermoFisher, Waltham, MA) with tandem Orbitrap Exploris 120 mass spectrometer (ThermoFisher, Waltham, MA). Reverse phase chromatography was performed using an Accucore biphenyl column (2.1 x 50 mm, 2.6 µm particle size; ThermoFisher, Waltham, MA) with 0.1% formic acid as mobile phase A and acetonitrile as mobile phase B. Flow rate was 0.4 mL/min and the solvent gradient for mobile phase B was as follows: 0 to 0.5 min held at 5%, 0.5 to 4.0 min increased from 5% to 95%, 4.0 to 4.75 min held at 95%, 4.75 to 5.5 min decreased from 95% to 5%, and then held at 5% to 6.5 min. Analysis was performed in positive ion mode with a full scan mass range of 175–300 m/z and mass resolution mode of 60K. Ionization was conducted using a heated electron spray ionization (HESI) source. XCalibur (ThermoFisher, Waltham, MA) software was used to integrate and report peak area for the M+H ions for DOM (210.1 m/z at a retention time of 3.85 min), DOPR (238.2 m/z at a retention time of 4.25 min), DOBU (252.2 m/z at a retention time of 4.4 min), DOAM (266.2 m/z at a retention time of 4.5 min), and 2,5-DMA (196.1 m/z at a retention time of 3.55 min), and to plot and fit a standard curve and to interpolate unknown values. For values above 500 ng/mL, samples were 1/10 or 1/20 diluted with 50% methanol containing 0.1% formic acid and reanalyzed.

3.5 Bioanalysis of microdialysis samples

A high-performance liquid chromatograph (Shimadzu, Kyoto, Japan) coupled with an API 5000 triple quadrupole tandem mass spectrometer (AB Sciex, Ontario, Canada) was used for the analysis. Summarized, the analytes were separated using a Kinetex 2.6 µm XB-C18 analytical column (100 Å, 50 mm × 2.1 mm; Phenomenex, Torrance, CA, USA); a mobile phase gradient of water and methanol supplemented with 0.1% formic acid was used. The analytes were detected in positive ionization mode using the following transitions: m/z 210.3 $[M+H]^+$ ➞ 178.2 (DOM); m/z 266.2 $[M+H]^+$ ➞ 234.2 (DOAM). DOM and DOAM levels were assessed in the range of 0.08–334 ng/mL and 0.04–334 ng/mL, respectively.

3.6 Molecular modeling

All modeling was conducted in Schrodinger Suite for small molecules (Schrödinger Release 2024-3; Schrödinger, LLC, New York, NY, USA). First, 3D ligand structures of studied ligands were built using Maestro modeling environment. LigPrep program was used to process ligands (assign protonation states at target pH of 7.4 +/- 1.0, enumerate chiral centers) for further studies. Next, conformational search in MacroModel using the OPLS4 force field in water was performed to localize global minimum structures. These were used as input for calculation of molecular descriptors using QikProp and for molecular docking using Glide program.

Protein structures of target receptors (for 5-HT_{2A} PDB IDs: 7WC4, 7WC8, 9AS8; for 5-HT_{2C} PDB ID: 8DPF) were downloaded from the PDB and fully preprocessed by Protein Preparation Wizard. Missing residues and atoms were reconstructed, and protonation states were assigned (corresponding to pH 7.4). The H-bonding network was fully optimized and finally the structure was minimized using the OPLS4 force field with the restricted minimization protocol (default settings). Resulting ligand-receptor complexes were then used for preparing the docking grids for Glide.
